# Probiotics and human health: biological activities, nutritional aspects, immunomodulatory properties, applications, and future perspectives - a comprehensive review

**DOI:** 10.3389/fimmu.2025.1713426

**Published:** 2026-03-24

**Authors:** Mohamed T. El-Saadony, Ahmed M. Saad, Mahmoud Sitohy, Samar Sami Alkafaas, Mthokozisi Dladla, Soumya Ghosh, Dina Mostafa Mohammed, Essam H. Ibrahim, Mohamed A. Fahmy, Amr Elkelish, Synan F. AbuQamar, Khaled A. El-Tarabily

**Affiliations:** 1 Department of Agricultural Microbiology, Faculty of Agriculture, Zagazig University, Zagazig, Egypt; 2 Biochemistry Department, Faculty of Agriculture, Zagazig University, Zagazig, Egypt; 3 Molecular Cell Biology Unit, Division of Biochemistry, Department of Chemistry, Faculty of Science, Tanta University, Tanta, Egypt; 4 Human Molecular Biology Unit, School of Biomedical Sciences, Faculty of Health Sciences, University of the Free State, Bloemfontein, South Africa; 5 Natural and Medical Sciences Research Center, University of Nizwa, Nizwa, Oman; 6 Department of Research Development, University of the Free State, Bloemfontein, South Africa; 7 Nutrition and Food Sciences Department, National Research Centre, Giza, Egypt; 8 Biology Department, Faculty of Science, King Khalid University, Abha, Saudi Arabia; 9 Biology Department, College of Science, Imam Mohammad Ibn Saud Islamic University (IMSIU), Riyadh, Saudi Arabia; 10 Department of Biology, College of Science, United Arab Emirates University, Al Ain, United Arab Emirates

**Keywords:** alleviating mechanism, gut microbiota, health benefits, live biotherapeutics, next generation probiotics, probiotics

## Abstract

Probiotics, defined as living microorganisms, are widely recognized for their ability to positively influence the gut microbiota, an effect increasingly linked to a wide array of health benefits. They are claimed to treat or prevent conditions ranging from infant colic to cardiovascular disease, respiratory infections, and certain cancers. Since the beginning of the 21st century, consumer demand for probiotic-enriched foods has risen significantly, propelled by these health assertions. The consumption of such products has been associated with the alleviation of disorders, including irritable bowel syndrome, lactose intolerance, gastroenteritis, obesity, chronic diarrhea, allergies, atopic dermatitis, and infectious diseases. Recent advancements in microbiome and microbiota research are fundamentally transforming probiotic science. Cutting-edge studies on novel strains, their mechanisms, and potential applications are expected to revolutionize our understanding of their roles in human nutrition and medicine. Nevertheless, despite extensive research efforts, critical gaps remain regarding strain-specific mechanisms, optimal dosages, long-term safety, and interactions among probiotics, host genetics, and dietary factors. Addressing these gaps necessitates a comprehensive synthesis of current knowledge and emerging trends. This review aims to critically integrate historical foundations, dosage strategies, mechanisms of action, therapeutic applications, and potential risks associated with probiotics. Unlike previous reviews, this review emphasizes next-generation probiotics, live biotherapeutics, and genetically engineered microbes, and their synergistic interactions with dietary bioactives such as polyphenols and fibers. By providing a forward-looking perspective, this work contributes to the rational design of functional foods, targeted therapies, and microbiome-based interventions, thereby informing future advancements in human nutrition and medicine. It critically examines current and emerging trends in probiotic research, while acknowledging potential adverse effects and risks.

## 1 Introduction

A major challenge of the 21st century is to sustainably nourish the rapidly growing global population amid increasing resource scarcity. Consequently, scientific research has increasingly emphasized the crucial role of a balanced diet in maintaining human health (1). A substantial body of evidence now suggests that regular consumption of probiotic-rich foods can reduce the incidence of various diseases (1).

However, reported benefits are not uniform across all trials or populations, and effect sizes are often modest, highly strain-specific, and influenced by background diet and host factors, which complicates broad generalization of these findings (1–3). This compelling evidence has spurred intensive research and development of novel natural products, fostering significant innovation in the food industry and stimulating the emergence of new functional food markets (1–3).

The gut microbiota, the intricate assembly of microorganisms inhabiting the gastrointestinal (GI) tract, assumes a critical role in essential physiological functions, including intestinal development, energy regulation, nutrient absorption, and immune modulation (4). Advances in microbiome research are opening innovative pathways for probiotic applications, with ongoing clinical studies anticipated to provide significant insights into their effects on human health (2). Nonetheless, many clinical investigations are limited by short follow-up durations, small sample sizes, heterogeneous formulations, and reliance on surrogate endpoints, such as variations in bacterial diversity or inflammatory markers, rather than definitive clinical outcomes, thereby limiting the robustness of current conclusions (2, 4).

This review is expected to clarify the mechanisms underlying the beneficial and nutritional functions of probiotics and to guide the development of sophisticated, next-generation probiotic (NGPs) formulations. A recent 2021 study by Cunningham et al. (5) emphasized that the advancement of microbiome-targeted interventions signifies a new epoch with transformative potential for healthcare.

Probiotic applications are increasingly guided by the principles of personalized nutrition, integrating nutrigenetics, nutrigenomics, and precision medicine (6). This forward-looking strategy seeks to tailor interventions to target microbial communities implicated in health and disease (7). Nutrigenetics examines how individual genetic variations predispose certain individuals to adverse responses to specific dietary components. In contrast, nutrigenomics investigates how bioactive nutrients modulate gene expression through activation, repression, or other regulatory mechanisms (5). Yet, truly personalized probiotic strategies are still at an early stage: only a minority of studies stratify outcomes by host genotype, baseline microbiome configuration, or dietary pattern, and inter-individual variability in response remains poorly understood (5, 7).

Human health is intimately linked to the diverse ecosystems of bacteria, fungi, viruses, and yeasts that coexist symbiotically on and within the body (8). Approximately 95% of these microorganisms reside in the GI tract, primarily in the colon, with smaller populations inhabiting the stomach and the small intestine (8). Genomic sequencing and advances in microbiology have yielded unprecedented insights into the functions and metabolites of these communities, driving the widespread adoption of the terms microbiota and microbiome in both scientific and industrial contexts (8).

In 2018, Dieterich et al. (7) defined microbiota as the complete community of microorganisms inhabiting a specific environment, such as the skin, oral cavity, lungs, urogenital tract, or gut. The term microbiome extends this concept to encompass the collective genetic material of these microorganisms, forming a dynamic and living ecosystem. Despite these conceptual advances, current microbiome profiling methods—often based on 16S rRNA sequencing—provide limited functional resolution, and only a subset of studies incorporates shotgun metagenomics or metabolomics to directly link microbial shifts to host pathways, leaving important mechanistic questions unresolved (7, 8).

It is noteworthy that the gut microbiota functions as a central mediator in the bidirectional communication between the GI system and the brain, a network commonly known as the microbiota–gut–brain axis (9). Deriving from the Greek term meaning ‘for life,’ the contemporary term ‘probiotic’ was first recorded by Ferdinand Vergin in 1954 (10).

The term experienced a semantic evolution in 1965, when Lilly and Stillwell redefined it to describe metabolites secreted by one microorganism that stimulate the growth of another (11). In 1974, Parker broadened the concept of probiotics to include any organisms or substances that help maintain intestinal microbial balance (12). Later, in 1989, Fuller narrowed this to “live microbial feed supplements” that benefit the host, effectively shaping the modern, health-focused definition and acknowledging their use across foods, supplements, and topical products (13).

A further key refinement in 1998 emphasized that such benefits occur only when probiotics are administered at sufficient doses (14). The most authoritative and contemporary definition was articulated in 2002 by a joint Food and Agriculture Organization (FAO) and World Health Organization (WHO) expert consultation, which described probiotics as “live microorganisms which, when administered in adequate amounts, confer a health benefit on the host” (15). This consensus was, in part, a response to an international trade dispute presented to the FAO/WHO by Argentina regarding the categorization of milk powder formulations containing viable lactic acid bacteria strains (16).

The panel concluded that probiotic efficacy is both strain-specific and dose-dependent, indicating that health benefits demonstrated by one strain cannot necessarily be generalized to others, even within the same species (17). This precise definition received formal endorsement from the International Scientific Association for Probiotics and Prebiotics (ISAPP) in 2014 (18). Individual probiotic strains possess distinct mechanisms of action and confer specific health benefits. Commonly utilized probiotics include selected strains from the genera *Lactobacillus*, *Bifidobacterium*, *Bacillus*, and *Pediococcus*, as well as certain yeast species (19).

Robust clinical evidence supports the use of specific probiotic strains in managing GI disorders such as irritable bowel syndrome (IBS), *Helicobacter pylori* infection, inflammatory bowel disease (IBD), and infectious diarrhea (2). Well-established benefits also extend to allergic conditions such as atopic dermatitis, metabolic disorders, including non-alcoholic fatty liver disease, obesity, insulin resistance, and type 2 diabetes; and certain cancers, where probiotics may help alleviate treatment-related adverse effects (3).

Simultaneously, several meta-analyses and guideline panels highlight that many claimed indications are supported only by low- to moderate-quality evidence, with significant heterogeneity between trials and frequent failure of some strains or formulations to demonstrate superiority over placebo in rigorous, well-controlled studies (2, 3). Ongoing research continues to explore their potential to enhance immune, metabolic, oral, and cognitive health, as well as their possible role as adjunctive therapy for COVID-19 (3).

This review examines the mechanisms of action of probiotics and summarizes current evidence regarding their effects on human health. Critical to this discussion is the emphasis on the careful selection of probiotic strains and the dosages required to elicit therapeutic effects, topics explored in dedicated sections. Further research is essential to discover new strains, optimize dosing regimens, confirm safety, and validate health claims through robust clinical trials.

Important gaps persist, including limited long-term safety data in vulnerable populations, incomplete monitoring of antimicrobial resistance transfer, and scarce head-to-head comparisons between strains or formulations, which hinder the development of clear, strain-specific clinical guidelines (3, 17). Despite rapid advances, critical challenges remain in probiotic research, including the strain-specific nature of effects, optimizing dosage strategies, validating long-term safety, and integrating with host genetics, diet, and microbiome diversity. Addressing these issues will require large, well-characterized cohorts, standardized outcomes, and multi-omics approaches that can connect microbial changes to host pathways and clinically meaningful endpoints (4, 8).

This review aims to (i) summarize current evidence on probiotic mechanisms and health benefits, (ii) critically evaluate therapeutic applications and associated risks, and (iii) highlight novel directions such as NGPs, engineered strains, and microbiome-targeted interventions. By offering a forward-looking synthesis, this work contributes to the rational design of functional foods and biotherapeutics, advancing both human nutrition and precision medicine.

## 2 History of probiotics

The functional concept of probiotics originates from ancient fermentation practices that predated the scientific discovery of microbes (19). Historical and archaeological evidence from ancient Egypt and India indicates that fermented dairy products and beverages were consumed for their perceived health-promoting properties, long before the responsible bacteria and yeasts were identified (19–21).

The modern scientific framework emerged in the early 20th century with Metchnikoff, who proposed that yogurt-derived bacteria could beneficially modulate the gut microbiota and improve health, opening the way for systematic probiotic research and strain isolation from diverse sources, including non-traditional niches such as fish gallbladders (21, 22). Today, probiotics are broadly grouped into *Lactobacillus*, *Bifidobacterium*, and several other genera. Lactobacilli, first described in 1901, are rod-shaped bacteria central to food fermentation, naturally inhabit the human GI and vaginal tracts, and provide both technological and health benefits when delivered in foods or supplements (22).

Genomic re-evaluation has recently split the former *Lactobacillus* genus into 25 genera, including the revised *Lactobacillus* and new genera such as *Lacticaseibacillus, Lactiplantibacillus*, and *Limosilactobacillus*, reflecting substantial phylogenetic diversity with clinical relevance (22). *Bifidobacterium* species, obligate anaerobes colonizing the distal small intestine and colon, support gut homeostasis by metabolizing dietary substrates, inhibiting pathogens, modulating inflammatory responses, maintaining barrier integrity, and contributing to micronutrient and bioactive compound production, with emerging evidence for antitumor potential (23, 24).

Other genera, such as *Enterococcus*, are exploited in food systems for their robustness, adhesion capacity, and bacteriocin production, which underpins both probiotic potential and natural biopreservation applications (25). The yeast *Saccharomyces cerevisiae*, particularly non-pathogenic probiotic strains, is widely employed as an adjunct therapy for GI disorders such as antibiotic-associated diarrhea; unlike many bacteria, these yeasts demonstrate high survival rates through the GI tract and can assist in stabilizing microbial balance and modulating immunity during infections or chronic illnesses (26, 27).

Probiotic efficacy is generally measured in colony-forming units (CFU), with typical daily doses ranging from 1 × 10^9^ to 1 × 10¹^0^ CFU, though clinical benefits are highly dependent on the specific strain or combination of strains, as many effects—such as the prevention of diarrheal diseases—are strictly strain-specific (28).

Concurrently, accumulating evidence suggests that several core mechanisms, namely, pathogen inhibition, enhancement of epithelial barrier function, and immunomodulation, are common across multiple taxa, providing a mechanistic foundation for the extensive commercialization of probiotics in dietary supplements, functional foods and beverages, and topical cosmetic formulations worldwide (28).

## 3 Key probiotic microorganisms and their functional characteristics

Key probiotic microorganisms in the gut are typically Gram-positive or yeast species that can survive GI transit, transiently colonize the intestine, and interact with the resident microbiota and host tissues (19). Collectively, these microbes contribute to carbohydrate fermentation by producing organic acids, support mineral absorption and bioavailability, help maintain epithelial barrier integrity, and modulate immune and metabolic pathways linked to inflammation, infection, and lipid homeostasis (29). When administered in adequate amounts, they form the basis of food and supplement formulations designed to inhibit pathogen overgrowth, reduce diarrheal and GI symptoms, and promote overall gut and systemic health (19, 29).

**Figure 1:**
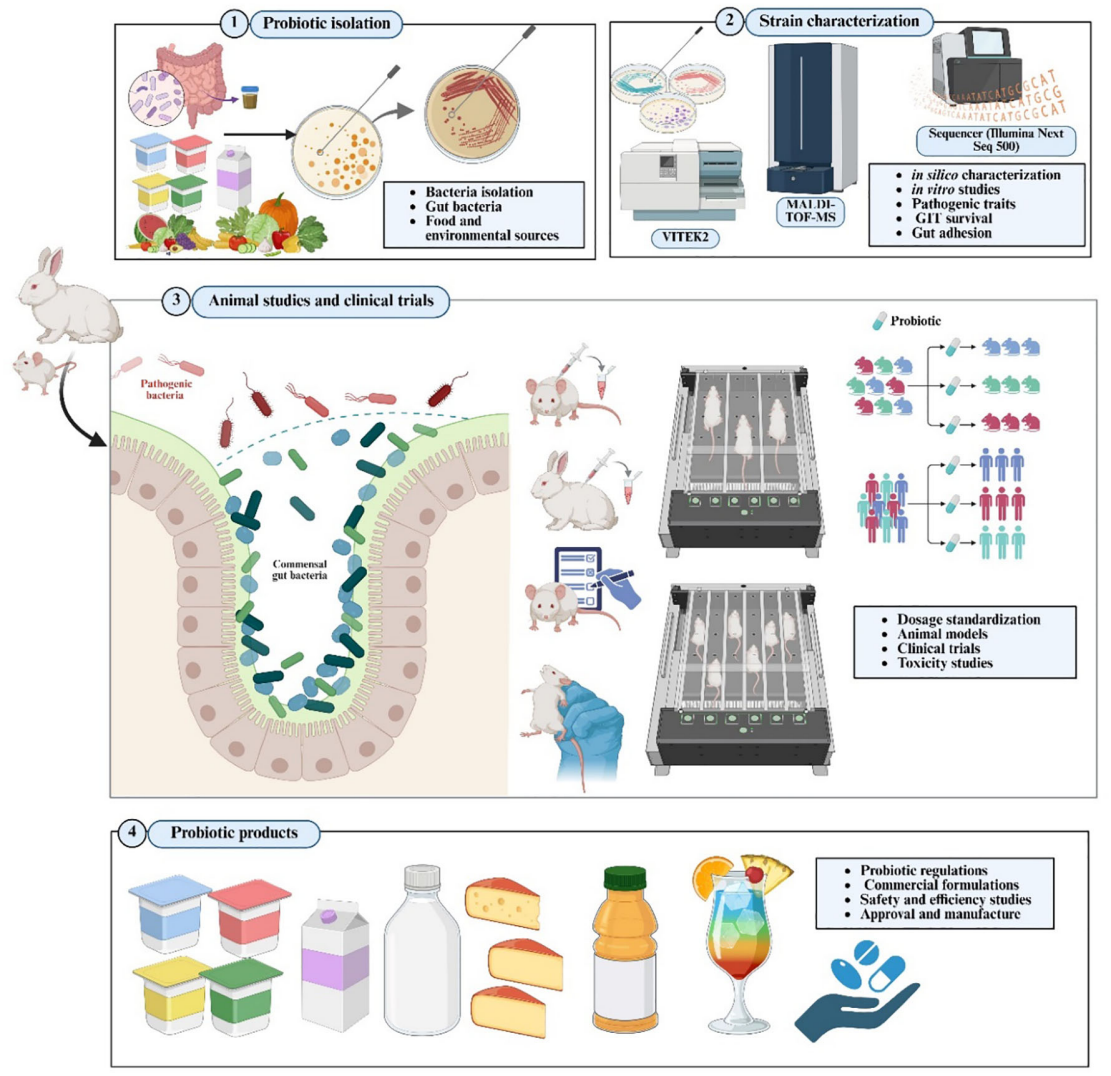
The translational pipeline of probiotics: from strain isolation and characterization to industrial commercialization. MALDI-TOF MS, matrix-assisted laser desorption ionization-time of flight mass spectrometry; GIT, gastrointestinal tract.

**Figure 1** illustrates the pathway from the isolation of probiotic strains to industrial-scale production. The strains listed in **Table 1** are exemplary microorganisms that have successfully progressed through this procedure. These include *Lactobacillus rhamnosus* GG, *Lacticaseibacillus casei*, *Bifidobacterium longum*, as well as next-generation taxa such as *Akkermansia muciniphila* and *Faecalibacterium prausnitzii.* These strains demonstrate how rigorous taxonomic characterization, systematic functional screening, and comprehensive safety evaluation can be combined to develop probiotic products for clinical use. Their use is consistent with FAO/WHO and subsequent guideline recommendations (29, 48). The following are some examples of beneficial gut probiotics.

**Table 1:** Mechanisms of action and clinical efficacy of major probiotic strains in human health.

Probiotic strain	Primary mechanisms of action	Key host outcomes	Primary application areas	References
*Lactobacillus rhamnosus* GG	Epithelial barrier enhancement; immune modulation (IgA, Treg); competitive exclusion	Reduced diarrhea risk; improved barrier function; reduced infection susceptibility; enhanced immune response; IgA production	General health; diarrhea prevention; GI disorders; immune support	(30, 31)
*Lacticaseibacillus casei*	Bacteriocin production; immune modulation; SCFA production; adhesion to epithelium	Improved intestinal health; reduced inflammation; enhanced microbiota stability; symptom relief in GI disorders	Digestive health; immune modulation; general probiotic support	(32)
*Lactiplantibacillus plantarum*	Broad SCFA production; immune tolerance; bacteriocin synthesis; anti-inflammatory effects	Enhanced barrier integrity; reduced pathogenic colonization; systemic immune support; improved metabolic markers	Barrier function; systemic inflammation; immune support; metabolic health	(33, 34)
*Bifidobacterium longum*	Immune modulation (Treg induction); metabolite production; microbiota modulation; epigenetic regulation	Reduced systemic inflammation; immune homeostasis; improved gut comfort; enhanced metabolic status; Treg expansion	Immune tolerance; metabolic health; general microbiota balance	(35)
*Bifidobacterium breve*	Metabolite production; immune tolerance; microbiota modulation; barrier support	Lower inflammation; microbiota stability; barrier support; safe for vulnerable populations; immune tolerance	Infant and child health; microbiota establishment; barrier development	(36)
*Bifidobacterium animalis* subsp. *lactis*	Immune modulation; SCFA production; metabolite signaling; anti-inflammatory effects	Anti-inflammatory effects; reduced infection risk; barrier stability; metabolic support; immune cell conditioning	Immune health; general wellness; metabolic support; barrier integrity	(37)
*Streptococcus thermophilus*	Lactose fermentation support; enzyme production; immune modulation; minor metabolite contribution	Enhanced lactose tolerance; nutrient absorption support; mild immune modulation; generally recognized as safe	Yogurt applications; lactose tolerance; general digestive health	(38)
*Saccharomyces cerevisiae* var. *boulardii*	Toxin neutralization; barrier support; competitive exclusion; antimicrobial peptide production	Protection from antibiotic-associated diarrhea (AAD); toxin neutralization; barrier repair; reduced pathogenic colonization	Antibiotic-associated diarrhea (AAD); *Clostridioides difficile* prevention; barrier repair	(39)
*Bacillus coagulans*	Spore survival; antimicrobial enzyme production; immune support; competitive exclusion	Immune support; spore stability; reduced infection risk; systemic immune activation; anti-inflammatory effects	GI health; spore stability; thermal stability; immune support	(40, 41)
*Bacillus subtilis*	Enzyme and metabolite production; antimicrobial effects; biofilm formation; sporulation advantage	Enhanced enzyme availability; antimicrobial activity; immune support; spore survival through harsh conditions; biofilm benefits	Enzyme supplementation; antimicrobial support; environmental tolerance	(42, 43)
*Akkermansia muciniphila*	Mucus layer enhancement (mucin degradation); barrier integrity; SCFA/propionate production; lipid/bile acid metabolism; epigenetic regulation (histone acetylation)	Improved metabolic health (glucose homeostasis, lipid profile reduction); enhanced barrier integrity (ZO-1, occludin, claudin-3 upregulation); reduced insulin resistance; decreased adipose tissue inflammation; reduced metabolic endotoxemia; epigenetic homeostasis	Metabolic disorders (obesity, T2DM, metabolic syndrome); barrier integrity; liver health; systemic inflammation	(44)
*Faecalibacterium prausnitzii*	Potent butyrate production; anti-inflammatory signaling (Dact3, NF-κB suppression); barrier reinforcement; IL-8 reduction; bile acid metabolism	Attenuated intestinal inflammation (IL-8 reduction, NF-κB suppression); improved barrier function; reduced inflammatory bowel disease symptoms; metabolic health (butyrate-mediated glucose regulation); anti-inflammatory cytokine production; protective in T2DM	Inflammatory bowel disease; inflammatory bowel disease metabolic disease (T2DM); barrier dysfunction; systemic inflammation; atherosclerosis prevention	(45)
*Roseburia faecis*	Butyrate production (prominent); barrier integrity; anti-inflammatory effects; immune modulation; microbiota stabilization	Enhanced gut barrier integrity; anti-inflammatory state; reduced intestinal permeability; improved butyrate availability; reduced risk in ulcerative colitis and metabolic disorders; T cell immune regulation	Type 2 diabetes; ulcerative colitis; metabolic disease; barrier support; anti-inflammatory conditions	(46)
*Propionibacterium freudenreichii*	Propionate production; extracellular vesicle (EV) signaling; NF-κB pathway modulation; immune tolerance; anti-inflammatory effects	Systemic immune modulation via EVs; NF-κB pathway suppression; reduced chronic inflammation; improved barrier function; anti-inflammatory cytokine upregulation; potential adjunct in immune-related conditions	Systemic inflammation; immune dysregulation; liver health; barrier dysfunction; general inflammatory disorders	(47)

AAD, antibiotic-associated diarrhea; IgA, immunoglobulin A (sIgA, secretory immunoglobulin A); IL, interleukin; SCFA, short-chain fatty acid; T2DM, type 2 diabetes mellitus; Tregs, regulatory T cells. EV, extracellular vesicle; GI, gastrointestinal tract; Dact3, dishevelled binding antagonist of beta-catenin 3; NF-kB, nuclear factor kappa B.

### 3.1 *Lactobacillus* and *Lactococcus* species: therapeutic profile

The genera *Lactobacillus* and *Lactococcus* are cornerstones of probiotic research, characterized by diverse physiological traits and broad-spectrum clinical applications (**Table 2**).

**Table 2:** Examples of key probiotic species, their mechanisms of action, and clinical applications.

Genus	Species/Strain	Key physiological traits	Mechanisms of action	Clinical and therapeutic applications	References
***Bifidobacterium***	*B. breve*	Commensal; dominant in breastfed infants; human milk resident.	Early gut colonization; competitive exclusion.	Pediatric GI disorders (diarrhea); respiratory infections (flu, cold).	(49–51)
	*B. longum* subsp. *infantis*	Gram-positive; high abundance in the infant gut. Strain: 35624.	Cytokine normalization (Anti-inflammatory/Pro-inflammatory ratio).	IBS treatment; alleviates visceral pain, bloating, and distension.	(52, 53)
	*B. longum*	Dominant gut resident; bioactive metabolite synthesis.	Mucin degradation (barrier function); host receptor interaction.	Antibiotic-associated diarrhea; ulcerative colitis remission; cancer inhibition (murine).	(54, 55)
	*B. lactis*	Robust stability in lyophilized (powder) form and food matrices.	Immune modulation; enhancement of digestive enzymes.	General GI health; immune response modulation (Strain HN019).	(56)
	*B. thermophilum*	Aerotolerant; survives low oxygen; produces BLIS.	Bacteriocin production (BLIS) targeting specific pathogens.	Inhibition of *Listeria*, *Salmonella*, *Campylobacter jejuni*, and rotavirus.	(57, 58)
***Lactobacillus***	*L. acidophilus*	Acid-tolerant; synergistic in multi-strain blends.	Cholesterol assimilation; gut barrier reinforcement.	Cardiovascular; cholesterol reduction (>50%); Cold symptom reduction (pediatric).	(19, 59)
	*L. rhamnosus*	Extreme pH tolerance; crucial for cheese organoleptics.	Metabolic fermentation; immunomodulation.	IBS; eczema; allergic disorders; flavor development in dairy.	(60)
	*L. fermentum*	High auto-aggregation; antioxidant properties.	Macrophage activation, IgA upregulation, and adhesion.	Cognitive benefits, anti-infective, and cholesterol metabolism.	(61)
	*L. johnsonii*	High bile and antibiotic tolerance. Strain: LA-1.	Competitive exclusion of pathogens; biofilm inhibition.	*Helicobacter pylori* gastritis; MDR organism antagonism; diarrhea duration reduction.	(62, 63)
	*L. reuteri*	Broad-spectrum antimicrobial.	Reuterin production; inhibition of P-selectin adhesion.	Infantile colic; dental caries; urogenital health; colitis.	(64)
***Lactococcus***	*L. lactis*	Gastroduodenal stress tolerance. Strain: ML-2018.	Downregulation of LPS-induced cytokines (TNF-α, IL-6).	IBD symptom alleviation; respiratory infection prevention.	(65, 66)
***Bacillus***	*B. coagulans*	Spore-forming; heat and acid-resistant.	Dormant gastric transit; germination in the intestine.	Functional foods (cooking stable); GI homeostasis; pathogen suppression.	(67)
***Streptococcus***	*S. thermophilus*	Dairy starter; lactose-hydrolyzing.	Proto-cooperation (Synergy) with bifidobacteria.	Lactose intolerance; rotavirus prevention (when combined with *Bifidobacterium bifidum*).	(68)
***Enterococcus***	*E. faecium*	Non-transferable resistance (safe strains); bile-tolerant.	Enterocin production (Class II bacteriocins).	Veterinary and livestock diarrhea; *Listeria* control; competitive exclusion.	(69)
***Saccharomyces***	*S. boulardii*	Yeast (Eukaryotic); antibiotic resistant.	Toxin cleavage; proteolytic digestion of *Clostridioides difficile* toxins A or B.	*Clostridioides difficile* infection; antibiotic-associated diarrhea; IBD.	(70, 71)

GI disorders, gastrointestinal disorders; IBS, irritable bowel syndrome; BLIS, bacteriocin-like inhibitory substances; MDR, multidrug-resistant organism; LPS, lipopolysaccharide; TNF-a, tumor necrosis factor alpha; IL-6, Interleukin-6; IBD, inflammatory bowel disease; GI (homeostasis), gastrointestinal; IgA, immunoglobulin A.

#### 3.1.1 Lactobacillus acidophilus

A leading probiotic validated for its ability to reduce cholesterol by over 50% *in vitro*, making it a valuable adjunct for cardiovascular wellness (72). Clinically, strains such as SBT-2026 and NCFM demonstrated efficacy in preventing GI infections in adults and reducing common cold severity in children (19, 59).

#### 3.1.2 L. rhamnosus

Distinguished by its tolerance to extreme GI pH gradients, this species is also industrially significant for driving organoleptic changes in cheese maturation (73). Therapeutically, specific strains mitigate pathologies including IBS, eczema, and allergic disorders, while eliciting broad immunomodulatory effects (60).

#### 3.1.3 Lactobacillus fermentum

Characterized by high auto-aggregation and adhesive capacity, strain JDFM216 exhibits strong anti-infective and antioxidant properties (74). It promotes physiological wellness through immunomodulation—specifically upregulating immunoglobulin (IgA) and macrophage activity—and supports cardiovascular health via cholesterol metabolism (61).

#### 3.1.4 Lactobacillus johnsonii

Exemplified by the seminal strain LA-1, this species is noted for its resilience to bile and antibiotics (75). Its efficacy involves the competitive exclusion of pathogens and direct antagonism of multidrug-resistant organisms, clinically correlating with reduced durations of diarrheal diseases and enterocolitis (62, 63).

#### 3.1.5 Lactococcus lactis

A primary species in probiotic research, specific strains like ML-2018 demonstrated potent anti-inflammatory activity by downregulating LPS-induced cytokines (65, 66). It is effective in alleviating IBD symptoms and reducing the risk of respiratory and GI infections (76).

#### 3.1.6 Lactobacillus reuteri

This species offers a broad therapeutic spectrum, ranging from urogenital health and dental caries prevention to the management of infantile colic (77). It promotes homeostasis by inhibiting P-selectin-mediated inflammation and exhibiting antimicrobial activity against enteropathogens and fungi (64).

### 3.2 *Bifidobacterium* species: therapeutic and functional profiles

The genus *Bifidobacterium* comprises a diverse group of probiotic bacteria critical for maintaining GI homeostasis and developing functional therapeutic applications (**Table 2**).

#### 3.2.1 Bifidobacterium breve

As a key commensal representative, *B. breve* is integral to the infant gut microbiota and is naturally present in human milk, facilitating early microbial colonization (49–51). Clinical evidence supports its pediatric utility in managing systemic and GI disorders, including respiratory infections and diarrheal diseases (76).

#### 3.2.2 *B. longum* subsp. *infantis*

A vital Gram-positive agent for microbiome balance, this subspecies has demonstrated robust efficacy in clinical trials (78). Specifically, the strain 35624 significantly alleviates core IBS symptomatology, such as visceral pain and bloating, thereby improving quality of life in pediatric populations (52, 53).

#### 3.2.3 B. longum

Dominant in the infant gut, *B. longum* utilizes bioactive metabolites and host interactions to mitigate IBS symptoms and prevent pediatric antibiotic-associated diarrhea (54, 55). It further supports ulcerative colitis remission and intestinal barrier function via mucin degradation (79). Emerging research suggests additional roles in immunoregulation and carcinogenesis inhibition (52).

#### 3.2.4 Bifidobacterium lactis

Distinguished by its robust stability across diverse matrices and lyophilization, *B. lactis* is highly suitable for industrial formulation (56). Clinical studies indicated that strain HN019 significantly enhanced GI health and modulated immune responses (57).

#### 3.2.5 Bifidobacterium thermophilum

This aerotolerant species is characterized by its ability to proliferate in low-oxygen environments (58). It produces bacteriocin-like inhibitory substances (BLIS) with broad-spectrum efficacy against pathogens such as *Listeria* and *Salmonella*, underscoring its potential in advanced probiotic and functional food development (57).

Beyond *Lactobacillus* and *Bifidobacterium*, several other taxonomic groups demonstrate significant therapeutic and industrial value (**Table 2**).

### 3.3 Bacillus coagulans

A spore-forming bacterium distinguished by its exceptional resilience to high temperatures and gastric acidity, ensuring viable transit to the intestines (80). Clinically, it alleviates GI disturbances and modulates the microbiota to suppress enteric pathogens (**Table 2**). Its stability supports widespread application in functional foods like kimchi and yogurt (67).

### 3.4 Streptococcus thermophilus

A primary dairy starter culture essential for yogurt and cheese production, known for catalyzing lactose hydrolysis (81). Therapeutically, it modulates immune responses and, when combined with *Bifidobacterium bifidum*, significantly reduces the incidence of rotavirus-induced gastroenteritis in pediatric populations. It also shows promise in mitigating sepsis-mediated inflammation (68).

### 3.5 Enterococcus faecium

Selected strains subject to stringent safety oversight (ensuring no antibiotic resistance transfer) are noted for high tolerance to bile salts and gastric acid (82). These strains maintain microbiome balance by competitively excluding pathogens and are particularly effective in veterinary applications for controlling *Listeria* spp. and managing diarrhea (69).

### 3.6 S. cerevisiae

This multifunction yeast probiotic is distinct in its ability to bind and neutralize bacterial toxins (83, 84). It is highly effective in managing infectious diarrhea and IBD by enhancing secretory immunoglobulin A (SIgA) production and reinforcing gut barrier integrity (**Table 2**). Its strong autoaggregation and adherence properties further support its role in chemoprevention and DNA protection (70, 71).

The targeted application of these species underscores the genus’s collective utility in clinical and nutritional science. Their documented roles range from supporting early microbial colonization and mitigating chronic GI pathologies to enhancing immune function and providing biodefense against specific pathogens (70, 71).

Collectively, the robust evidence supporting the therapeutic, immunomodulatory, and industrial resilience of these strains confirms the critical value of the *Lactobacillus* and *Bifidobacterium* genera as cornerstones of NGPs development.

### 3.7 Mechanisms of action across probiotic genera

The therapeutic efficacy of the diverse probiotic spectrum, spanning *Bifidobacterium*, *Lactobacillus*, spore-formers, and yeasts, is underpinned by a convergent yet distinct set of molecular mechanisms that maintain host homeostasis.

#### 3.7.1 Immunomodulation and barrier reinforcement

A fundamental mechanism shared by *Lactobacillus* and *Bifidobacterium* species involves the modulation of host immunity through cytokine regulation (**Table 2**). Strains such as *B. longum* subsp. *infantis* 35624 and *L. lactis* actively downregulate pro-inflammatory signaling pathways (e.g., NF-κB) while upregulating anti-inflammatory cytokines such as IL-10. O’Mahony et al. (2005) demonstrated that *Bifidobacterium infantis* 35624 normalizes the ratio of IL-10 to IL-12 in patients with IBS, directly correlating with symptom alleviation (85). Similarly, *L. lactis* ML-2018 has been shown to inhibit nitric oxide release and suppress lipopolysaccharide (LPS)-induced inflammation in macrophage models (86).

Strengthening the intestinal epithelial barrier enhances this immunoregulatory effect. Species like *L. acidophilus* and *B*. *breve* boost mucin production and tighten junctions, effectively preventing “leaky gut” and subsequent systemic immune activation. In children*, L. acidophilus* NCFM has been shown to reduce fever and respiratory symptoms, indicating that reinforcing the gut barrier supports systemic immune defenses (87). Additionally*, B. longum* subsp. *infantis* can colonize the premature intestine and lower pro-inflammatory cytokines, offering protection against necrotizing enterocolitis (88).

#### 3.7.2 Direct pathogen antagonism and exclusion

Probiotics use various strategies to combat competitors. Competitive exclusion is exemplified by *L. johnsonii*, which blocks adhesion sites that pathogens require. Sgouras et al. (89) confirmed that *L. johnsonii* LA-1 antagonizes *H*. *pylori*, significantly lowering pathogen levels and associated gastritis. Chemical warfare is employed by species such as *L. reuteri* (which produces reuterin) and *E. faecium* (which produces enterocins), which disrupt pathogen membranes. Meta-analyses have verified *L. reuteri’*s effectiveness in treating infant colic, due to its broad-spectrum antimicrobial activity against gas-producing enteropathogens (90).

Additionally, safety-evaluated strains of *E. faecium* produce class II bacteriocins that effectively reduce *Listeria* populations (91). Uniquely, the yeast *Saccharomyces boulardii* works through enzymatic neutralization. Castagliuolo et al. (92) found that *S. boulardii* secretes a serine protease that directly cleaves *Clostridioides difficile* toxin A and toxin B, preventing enterotoxicity and receptor binding.

#### 3.7.3 Environmental resilience and metabolic synergy

Survival and delivery are optimized through distinct physiological traits. *B*. *coagulans* utilizes sporulation to remain dormant and resistant to gastric acidity until reaching the intestine. Majeed et al. (93) confirmed that *B. coagulans* MTCC 5856 spores survive gastric transit to significantly reduce diarrhea-predominant IBS symptoms (93).

Furthermore, metabolic synergy (protocooperation) enhances efficacy. *S. thermophilus* produces formate to stimulate *Bifidobacterium* growth. Saavedra et al. (94) provided seminal evidence that the specific combination of *B. bifidum* and *S. thermophilus* reduces the incidence of rotavirus shedding in hospitalized infants, validating the clinical relevance of this “consortium effect” (94).

**Figure 2:**
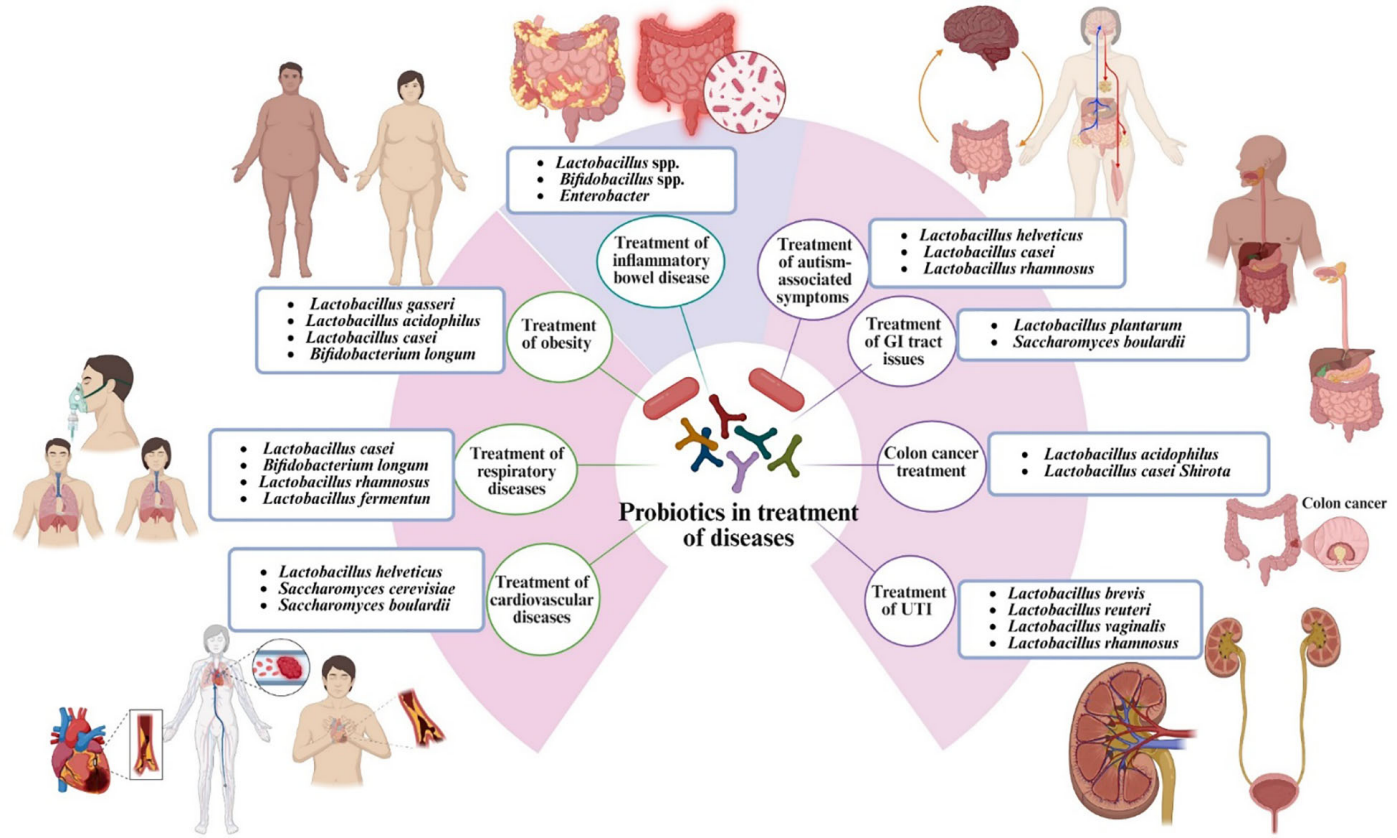
The therapeutic spectrum of probiotics: Strain-specific applications in the management of gastrointestinal and systemic disorders. GI, gastrointestinal; UTI, urinary tract infections.

**Figure 2** and **Table 1** summarize the primary disease domains influenced by probiotics, including GI, metabolic, infectious, oncological, and neuropsychiatric indications. Also, it refines this overview by aligning specific probiotic strains with these indications and delineating their predominant mechanisms of action. For example, *S. cerevisiae* var. *boulardii* and *L. rhamnosus* GG are predominantly associated with the prevention of antibiotic-associated diarrhea and *C. difficile* infection through mechanisms such as toxin neutralization, competitive exclusion, and barrier repair, agreeing with Uhegwu and Anumudu (95). Conversely, *F. prausnitzii*, *Roseburia faecis*, and *A. muciniphila* are more closely related to IBD, type 2 diabetes, obesity, and fatty liver disease primarily through the production of butyrate or propionate, suppression of NF-κB, and the enhancement of bile acid and lipid metabolism (96, 97).

## 4 Sources of probiotics: from traditional fermented foods to modern functional products

The growing global focus on health and wellness is driving demand for functional foods, products designed to provide targeted benefits, such as enhanced disease resistance and prevention, that extend beyond basic nutritional value (98). Fermented foods, which use live microorganisms during production, have a long history of use to improve digestive health, preserve food, and boost nutritional quality. Microbial consortia involved in fermentation and gut symbiosis are capable of synthesizing substantial amounts of vitamins B, C, and K, and actively participate in the breakdown of anti-nutritional compounds such as phytic acid, thereby increasing the bioavailability of essential compounds that would otherwise be poorly absorbed (99).

A key consideration is that modern industrial processing can adversely affect the survival of these beneficial microbes in the final product. Conversely, traditionally crafted fermented foods, including yogurts and pickles, are esteemed as valuable probiotic sources, with their positive health impacts substantiated in clinical studies (100). Probiotics are now chiefly marketed in two forms: as conventional foods and as dietary supplements, the latter of which falls under the regulatory authority of the Food and Drug Administration (FDA) (99, 101).

Although dairy remains the predominant vehicle for probiotics, incorporating them into non-dairy matrices, such as juices and plant-based beverages, is increasingly common. This diversification caters to both health-conscious consumers and the growing vegan demographic, which demands products free from animal ingredients. Moreover, inherent limitations of dairy-based products, including lactose content, cholesterol, and limited shelf life, are spurring innovation in the functional food industry. These challenges present key opportunities for product development, positioning the sector as an important arena for technological advancement and economic growth (101).

### 4.1 Cereal and dietary fiber matrices

Cereals serve as optimal probiotic substrates due to their complex carbohydrate profiles (starch, arabinoxylans, β-glucans) and nutrient density (102–104). Strains such as *Lactobacillus plantarum*, *L. acidophilus*, *L. reuteri*, and *L. fermentum* have been successfully cultivated on malt, barley, and wheat media (105).

Lactic acid fermentation in these matrices produces organic acids that enhance preservation and flavor. Furthermore, cereal-derived dietary fibers act as prebiotics; soluble fibers delay gastric emptying and modulate glucose absorption, while insoluble fibers support the structural integrity of the food matrix (106–108).

### 4.2 Vegetable and fruit vectors

Plant-based matrices offer protection against gastric acidity and provide intrinsic nutrients (fiber, antioxidants) that enhance probiotic viability (109, 110). Products like sauerkraut (*Leuconostoc mesenteroides*, *L. plantarum*) and kimchi (*Lactobacillus kimchii*, *Weissella* spp.) rely on sequential lactic acid bacteria colonization. These foods are correlated with immunomodulation, lipid regulation, and cardioprotective effects (111–114).

Also, *Citrus* and other fruit juices serve as non-dairy vehicles for *L. acidophilus* and *L. plantarum*. Probiotic fermentation in these matrices has been shown to increase the bioaccessibility of phenolic antioxidants (115, 116). Furthermore, in pickles, lactic acid fermentation of cucumbers by native microbiota ensures preservation and provides a source of vitamin K and live probiotics (117–119).

### 4.3 Dairy-based probiotic foods

Dairy remains the primary vehicle for probiotic delivery, leveraging established fermentation traditions. In yogurt and cheese, fermentation is driven by *Lactobacillus delbrueckii* subsp. *bulgaricus* and *S*. *thermophilus*, often fortified with *Bifidobacterium* and *L. acidophilus*. These products reduce the risk of osteoporosis, hypertension, and urogenital infections (120–124).

In Kefir and koumiss, these beverages use complex symbiotic cultures of bacteria (*Lactobacillus* spp.) and yeasts (*Candida* and *Kluyveromyces*). Koumiss (fermented mare’s milk) is noted for modulating immune responses and lowering cholesterol (125–128). In pediatric applications, the combination of *S. thermophilus* and *B. bifidum* has been correlated with reduced rotavirus-induced gastroenteritis (68).

### 4.4 Legume and soy fermentations

Traditional Asian fermentations use specific fungal and bacterial groups to improve bioavailability and produce bioactive metabolites. Soy products, such as miso and soy sauce, depend on *Aspergillus oryzae*, along with *Tetragenococcus halophilus* and *L. acidophilus*. These foods are associated with anti-diabetic, anti-hypertensive, and neuroprotective effects (129, 130).

Additionally, natto and tempeh involve *Bacillus subtilis* var. *natto* for natto fermentation, while *Rhizopus* converts soybeans into tempeh, creating protein-rich meat alternatives (131, 132). Several studies indicated that a high intake of fermented soy correlates with a lower risk of cardiovascular disease and hypertension (133, 134).

### 4.5 Novel and functional applications

Beyond nutrition, probiotics are increasingly integrated into functional beverages, cosmetics, and pharmaceuticals. Functional beverages like Kombucha (tea fermented by *Gluconacetobacter* and *Saccharomyces*) and Boza (millet) offer anti-inflammatory and antioxidant benefits (135–137).

The application of probiotics has expanded significantly beyond traditional nutrition, emerging as a bioactive frontier in dermatological and pharmaceutical sciences (137). In the cosmetic sector, formulations incorporating strains such as *B. subtilis*, *L. plantarum*, and *L*. *lactis* are engineered to adhere to the epidermal surface, where they inhibit pathogenic colonization and modulate local immunity (138, 139). These topical applications, ranging from serums to cleansers, demonstrate efficacy in managing dermatological conditions such as atopic dermatitis, acne, eczema, and psoriasis, as well as promoting tissue regeneration (138, 139).

Concurrently, the pharmaceutical industry has advanced the development of high-potency, multi-strain supplements (e.g., VSL#3) and functional non-dairy matrices (derived from oats, quinoa, and chia) to address specific clinical disorders and dietary restrictions like lactose intolerance. These innovations are supported by rigorous evaluations confirming their safety, viability, and therapeutic efficacy in both adult and pediatric populations (140, 141).

## 5 Cellular, molecular, and systemic mechanisms of probiotics

Recent advances in probiotic research have substantially refined the selection, genomic characterization, and functional assessment of specific microbial strains, leading to a deeper understanding of their mechanisms of action and their measurable impacts on human health (142). The therapeutic effects of probiotics are multifactorial and exhibit substantial interindividual variability, influenced by the composition and metabolic activity of the host’s resident gut microbiota, the specific probiotic strain or strain combination employed, and the precise site of action within the GI tract (143).

Key mechanisms through which probiotics exert beneficial effects include the reinforcement of the intestinal epithelial barrier by stimulating mucin protein synthesis and regulating tight junction proteins such as occludin and claudin-1 (143). Probiotics also improve mucosal adhesion, allowing them to competitively exclude pathogens by occupying binding sites on the gut lining and inhibiting pathogenic adhesion. Additionally, they secrete antibacterial compounds, such as organic acids, hydrogen peroxide, and bacteriocins, that reduce pathogen viability (144). Probiotics modulate the host immune system by enhancing the production of SIgA and anti-inflammatory cytokines, regulating macrophages, dendritic cells (DCs), and lymphocytes to maintain gut homeostasis and prevent infections (143–146).

In the GI environment, probiotics counteract harmful microorganisms by shortening GI transit time, limiting opportunities for pathogen colonization, and stimulating the production of bioactive metabolites, such as short-chain fatty acids (SCFAs), which lower colonic pH. Additional documented functions include the biosynthesis of vitamins, the metabolism and deconjugation of bile salts, diverse enzymatic activities that contribute to nutrient bioavailability, and the neutralization of dietary or microbial toxins (147).

Probiotics also support intestinal electrolyte absorption, influence lipid metabolism, and can bind directly to Gram-negative pathogens. A particularly important function is their ability to engage in biochemical signaling with host cells, exerting effects both locally within the intestinal mucosa and systemically through immune, neural, and metabolic pathways. This interaction reinforces the gut barrier and reduces pro-inflammatory cytokine secretion (148), and enhances immune responses (149).

The host’s innate intestinal defense system consists of a protective mucus layer and a specialized epithelial barrier, sustained by diverse, functionally distinct cell types. Paneth cells secrete antimicrobial peptides such as defensins in response to microbial stimuli, while *S*. *cerevisiae* has been shown to enhance mucin production and reinforce mucus secretion (150). Enterocytes facilitate nutrient absorption and maintain epithelial integrity, whereas enteroendocrine cells release regulatory peptides that contribute to barrier function and coordinate mucosal immune responses (151–153).

The mucus layer is vital for protecting intestinal tissues from luminal threats, facilitating nutrient transit, and preventing pathogen adhesion and invasion. By interacting with both indigenous microbiota and host intestinal cells, ingested probiotics strengthen the gut’s chemical, mechanical, biological, and immunological defenses (154).

Crucially, probiotics help restore normal intestinal permeability by promoting the regeneration and proliferation of mucosal epithelial cells. They stimulate mucus secretion, enhancing the protective mucosal layer, and support the structural integrity and functional maintenance of the mucosal barrier (155). Probiotics achieve these effects by upregulating tight junction protein expression, modulating epithelial cell apoptosis, and influencing immune signaling pathways to enhance barrier resilience. These actions collectively help maintain gut homeostasis, prevent pathogen translocation, and reduce inflammation (155). Through these coordinated actions, probiotics significantly improve health outcomes and mitigate disease in both pediatric and adult populations (155).

**Figure 3:**
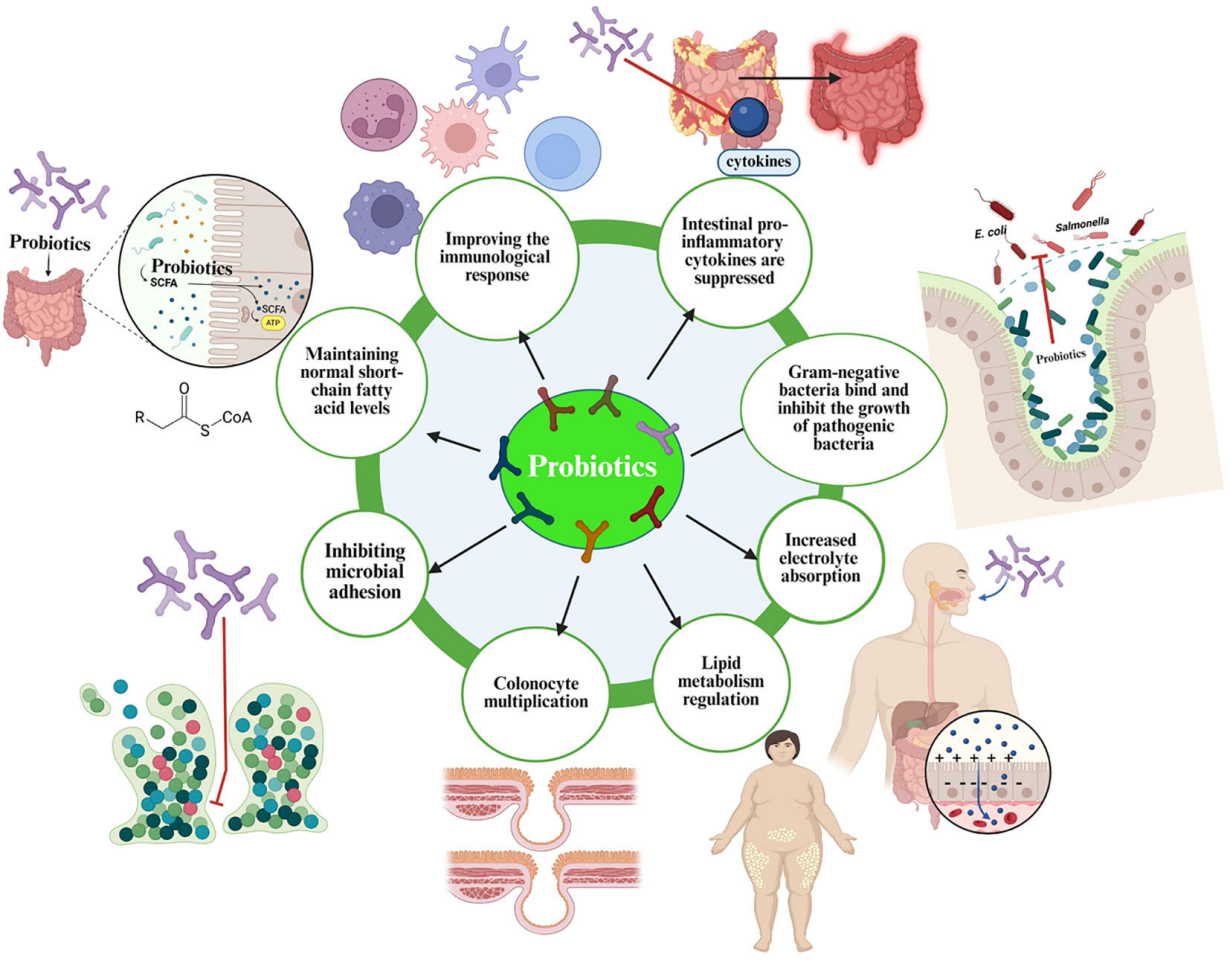
Multifaceted mechanisms of action underlying the therapeutic effects of probiotics. SCFA, short-chain fatty acid.

**Figure 4:**
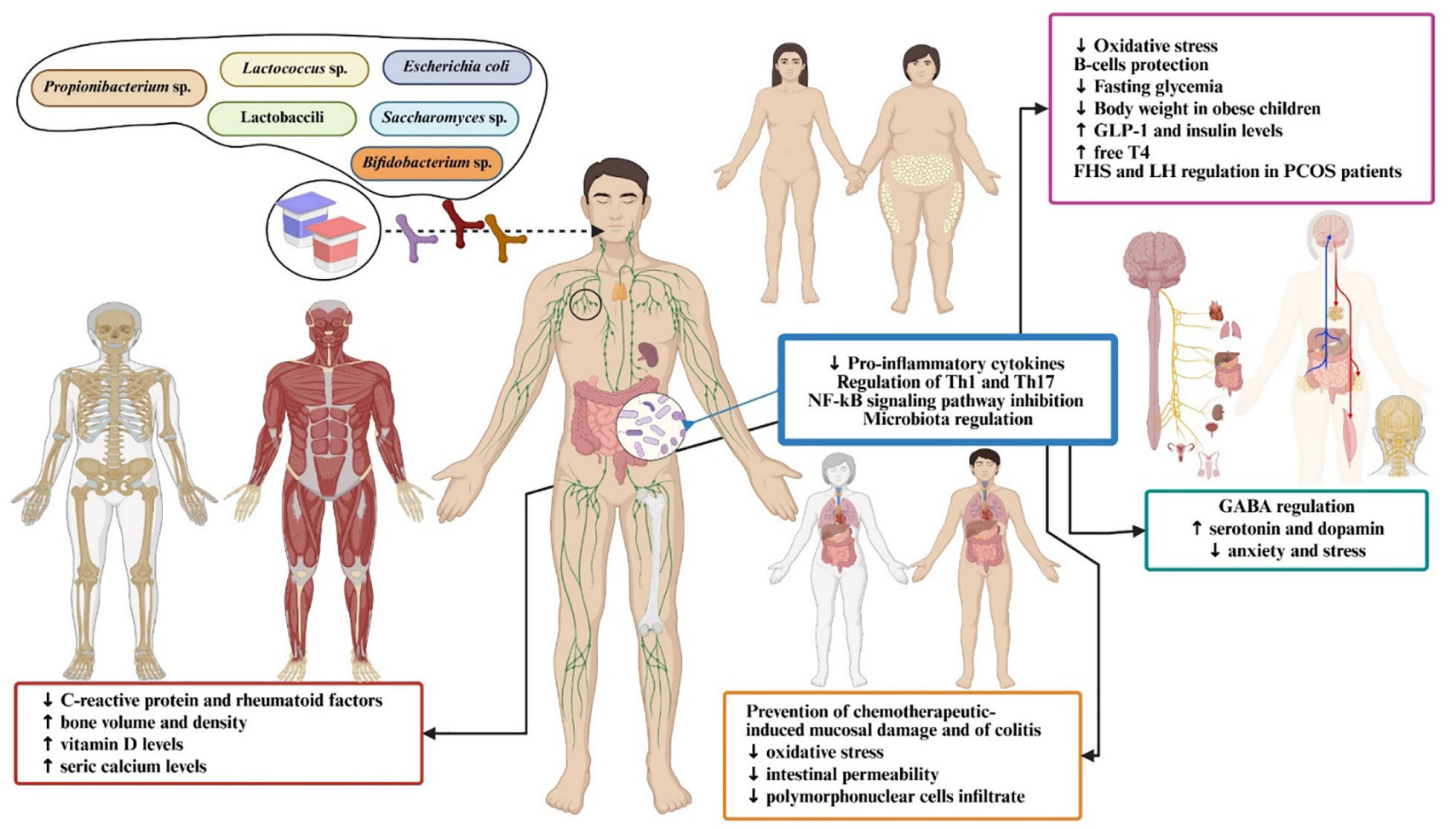
Physiological and metabolic impacts of probiotic administration across multiple organ systems. GABA, gamma-aminobutyric acid; GLP-1, glucagon-like peptide; FSH, follicle-stimulating hormone; LH, luteinizing hormone; PCOS, polycystic ovary syndrome; NF-kB, nuclear factor kappa B.

**Figures 3** and **4** depict the core mechanistic pathways and their contributions to systemic inflammation, which is supported by **Table 1**, which anchors these generic pathways in well-characterized strains. Barrier enhancement and tight-junction upregulation are exemplified by *L. rhamnosus* GG, *A. muciniphila*, and butyrate producers such as *F. prausnitzii* and *Roseburia* spp.

The direct antimicrobial and competitive effects of these strains are dominated by bacteriocin-producing lactobacilli and spore-forming *Bacillus* species (29, 156). Also, SCFA- and other metabolite-driven immune modulation, including histone deacetylase (HDAC) inhibition. Regulatory T cells induction, and cytokine rebalancing, is most prominent for butyrate and propionate producers (*F. prausnitzii*, *R. faecis*, *Propionibacterium freudenreichii*, *A. muciniphila*), which helps explain their impact on systemic inflammatory markers and cardiometabolic risk (157).

### 5.1 Probiotics and intestinal barrier protection

The intestinal epithelium functions as a critical first line of defense, preserving epithelial integrity and shielding the host from pathogenic microorganisms (158). This barrier function is maintained by multiple interdependent mechanisms, including the secretion of IgA, the production of a protective mucous layer, the formation of tight and adherens junctions, and the release of antimicrobial peptides. Disruption of this barrier permits microbial translocation into the submucosa, triggering inflammatory cascades that contribute to the pathogenesis of intestinal disorders such as IBD (159).

Administration of beneficial probiotic bacteria has been demonstrated to strengthen and maintain this essential intestinal barrier function (160). Emerging evidence indicates that specific probiotic strains can actively promote the restoration of epithelial barrier integrity after injury.

*In vitro* studies employing Caco-2 and T84 cell models have demonstrated that *Escherichia coli* Nissle 1917 (EcN1917) counteracts mucosal disruption induced by enteropathogenic *E. coli* and accelerates the reestablishment of epithelial integrity. This reparative effect is mediated, at least in part, through the upregulation and reorganization of key regulatory proteins, most notably protein kinase C (PKC) and the tight junction scaffolding protein zonula occludens-2 (ZO-2), thereby facilitating the reassembly and functional recovery of the tight junction complex (161).

Through enhancement of the mucosal barrier, probiotics help prevent chronic inflammation and increase the host’s resistance to diverse infectious agents (162). Their protective functions include reducing paracellular permeability, boosting innate immune responses against pathogens, and consolidating the physical barrier provided by the mucous layer (163). Collectively, these mechanisms enable probiotics to counteract intestinal barrier dysfunction associated with enteric infections, thereby supporting epithelial integrity and limiting pathogen-induced inflammation.

### 5.2 Cellular crosstalk

Intestinal epithelial cells (IECs) constitute a critical physical barrier that segregates the intestinal lumen, with its diverse contents, including digested nutrients, commensal microbiota, and potential pathogens, from the underlying mucosal immune system (164). The stability of this barrier depends critically on the integrity of tight junctions. These specialized protein complexes, composed of claudin, occludin, and zonulin, among other molecules, form a seal in the paracellular space between adjacent IECs, thereby tightly controlling the passage of ions and solutes (165). The organization of these transmembrane proteins is regulated by specific signaling cascades, such as the mitogen-activated protein kinase (MAPK) pathway (165).

Probiotics contribute to the preservation of tight junction function and gut barrier stability through several mechanisms. A principal mechanism of action involves the modulation of gene and protein expression governing tight-junction signaling pathways within intestinal IECs, thereby influencing barrier assembly, permeability, and overall epithelial integrity (166).

For instance, studies demonstrated that *L. plantarum* activates toll-like receptors (TLRs) signaling, leading to the translocation of the pivotal structural protein Zonula occludens-1 (ZO-1) to the tight junction domain in intestinal models (165). Additionally, probiotics can facilitate recovery of epithelial barrier integrity following injury from enteric infections or inflammatory conditions by promoting the apoptosis of damaged IECs. This controlled elimination of compromised cells aids their clearance and fosters subsequent mucosal repair and regeneration (167).

### 5.3 SCFA production and anti-inflammatory effects

Certain probiotic strains, particularly members of the *Bifidobacterium* and *Lactobacillus* genera, produce SCFAs, notably acetate, propionate, and butyrate, via the fermentation of dietary fibers (168). Among these metabolites, butyrate plays a key role in maintaining intestinal barrier integrity by strengthening tight junction architecture and modulating its regulatory signaling pathways (168).

The research by Peng et al. (169) demonstrated that butyrate enhances AMP-activated protein kinase (AMPK) activity in Caco-2 cell monolayers, thereby accelerating tight junction assembly and fostering a more resilient intestinal barrier. This SCFA-mediated reinforcement of intercellular junctions reduces the risk of increased intestinal permeability, commonly referred to as “leaky gut,” a pathological condition that enables the translocation of luminal antigens and other deleterious molecules into the systemic circulation (170).

Butyrate also provides direct anti-inflammatory benefits by inhibiting key pro-inflammatory signaling pathways, thereby counteracting cytokine-driven disruption of tight junction integrity. Through these mechanisms, SCFAs collectively suppress the production and expression of pro-inflammatory mediators, including tumor necrosis factor-α (TNF-α), interleukin-6 (IL-6), and nitric oxide, offering both direct and indirect protection of epithelial barrier function (171), while simultaneously promoting the synthesis and efficacy of the anti-inflammatory cytokine IL-10 (172, 173).

Probiotics play a crucial role in modulating prostaglandin E2 (PGE2), an anti-inflammatory prostanoid that inhibits the secretion of pro-inflammatory cytokines, such as IL-1β and TNF-α, by macrophages (174). Emerging evidence from recent *in vitro* studies and clinical trials indicates an inverse correlation between SCFA concentrations in the gut and the prevalence of disorders, including colorectal cancer, IBD, and diabetes. SCFAs produced by probiotic bacteria contribute to immune regulation by inhibiting inflammatory pathways, enhancing the production of anti-inflammatory cytokines, and maintaining intestinal barrier integrity. These metabolites also affect epigenetic and cellular mechanisms involved in tumor suppression and metabolic health, positioning SCFAs as key mediators in probiotic-induced health benefits (175–177).

Importantly, SCFA production is not limited to conventional probiotics; other commensal microbes, including *A. muciniphila* (178), *Bacteroides* spp. (179). *F*. *prausnitzii*, and *Clostridium symbiosum* (180, 181), are also major contributors. The collective activity of the gut microbiota is indispensable for maintaining intestinal homeostasis, a fundamental pillar of overall host health and a critical defense against a broad spectrum of pathological conditions (181).

### 5.4 Probiotic adhesion to the mucosa and host interaction

Probiotics play an important role in regulating the synthesis and structural composition of the protective mucus layer that coats the intestinal epithelium (117). A well-developed mucus layer is essential for maintaining appropriate host-microbiota interactions and for supporting the optimal function of epithelial tight junctions. A critical prerequisite for probiotics to exert these health-promoting effects is their ability to adhere to the intestinal mucosa, enabling transient colonization and effective biochemical communication with host cells (151, 182). This adhesive capability is fundamental for modulating immune responses and preventing pathogen attachment (182).

Probiotics, especially lactic acid bacteria, express specialized surface structures that facilitate interaction with IECs. These epithelial cells produce mucins, highly glycosylated proteins that constitute the main structural component of the intestinal mucus. This gel-like mucosal barrier serves as a primary defense mechanism by competitively inhibiting the adhesion of pathogenic bacteria to host surfaces (182, 183).

Specific molecular mechanisms underlie this process. For example, *L. reuteri* secretes a high–molecular–weight mucus-binding protein (MUB) that is pivotal for host attachment. This adhesion is mediated through interactions with mucosal lipid moieties and components of the bacterial cell wall, thereby stabilizing the probiotic’s association with the intestinal epithelium and facilitating sustained host–microbe communication (184, 185).

Beyond facilitating adhesion, probiotics can actively modulate mucin composition and glycosylation patterns, thereby enhancing the mucus layer’s capacity to impede pathogen attachment and colonization (183). Furthermore, probiotics enhance gut barrier integrity by stimulating epithelial cells to secrete antimicrobial proteins (AMPs), such as defensins, which exhibit broad-spectrum activity against bacteria, viruses, and fungi. Through the upregulation of these AMPs, probiotics make a substantial contribution to the stabilization and reinforcement of intestinal barrier function (165).

### 5.5 Probiotics in microbiome balance and dysbiosis prevention

Dysbiosis, an imbalance in the gut microbial community, is closely linked to the deterioration of intestinal barrier function (186). Probiotics support the restoration and maintenance of a balanced intestinal microbial ecosystem, which in turn strengthens the structural integrity of the gut epithelium and its tight junctions. Disruption of this homeostatic equilibrium can lead to dysbiosis and consequent metabolic perturbations that are increasingly implicated in the pathogenesis of chronic conditions such as cancer, cardiometabolic disorders, and IBD (187).

As reported by Blaabjerg and his co-workers, the ability of probiotics to preserve and restore gut homeostasis has been demonstrated in a wide range of disorders characterized by disrupted microbiota (188). A meta-analysis demonstrated that the concurrent administration of *L*. *rhamnosus* and *S. boulardii* was associated with a significant 51% reduction in the incidence of antibiotic-associated diarrhea in clinical trials, without any corresponding increase in adverse effects (188).

Supporting this, a study by Krebs (189) demonstrated that *Lactobacillus* probiotics induced substantial shifts in the gut microbiota of colorectal cancer patients, leading to improved mucosal structure correlated with enhanced colonization by *Lactobacillus* species. Further research is needed to fully elucidate the underlying mechanisms, as the cumulative body of evidence supports the potential of probiotics for both the prevention and management of human diseases associated with gut microbiome dysregulation.

### 5.6 Probiotics in the competitive exclusion of pathogens

Of particular interest in contemporary research is the capacity of probiotics to outcompete pathogenic bacteria (190). A growing body of evidence shows that probiotics participate in direct competitive interactions with pathogens, a process that is instrumental in sustaining a balanced gut microbiota and thereby supporting overall health. This competitive dynamic primarily revolves around the acquisition of essential resources, including nutrients and adhesion sites on the intestinal epithelium. By inhibiting the proliferation and colonization of harmful bacteria, probiotics foster a healthier microbial equilibrium and reduce susceptibility to infections (190).

For example, specific strains such as *Lactobacillus* GG and *Lactobacillus plantarum* 299V have been shown to competitively exclude *E. coli* by impeding its adhesion (191). Certain *Lactobacillus* species achieve this competitive advantage by occupying host receptor sites, thereby preventing pathogen adhesion. In addition, probiotic-derived metabolites are thought to reinforce this exclusion by modulating critical cell-signaling and metabolic pathways that influence epithelial receptivity and microbial colonization (192).

A critical step in the virulence of enteric pathogens is their adherence to intestinal epithelial cells. Probiotics counteract this mechanism by binding to these cells, either through electrostatic forces or via specialized surface adhesins. This capacity allows them to colonize adhesion sites, including colonic crypts, goblet cells, and intestinal villi, in substantial numbers, thereby physically preventing pathogens from gaining a foothold and establishing infection (163, 192). Research confirms that probiotics frequently demonstrate a superior capacity for epithelial adhesion compared to pathogens (193).

Illustrative examples include *L. reuteri* and *B*. *bifidum*, which adhere to host cell surface glycolipids, thereby effectively blocking pathogenic bacteria from binding to the same receptors and preventing their colonization (163). In essence, this process of competitive exclusion reflects a dynamic microbial interplay in which beneficial and pathogenic bacteria compete for mucosal binding sites and limited nutrient resources (194).

### 5.7 Antimicrobial compounds secreted by probiotics

A key mechanism by which probiotics exert their beneficial effects is through the secretion of antimicrobial compounds that inhibit the growth of pathogenic bacteria (195). These metabolites, including acetic acid, lactic acid, formic acid, and other SCFAs, reduce intracellular pH, creating an acidic microenvironment that is hostile to pathogen growth and proliferation (196). Additionally, numerous probiotics produce bacteriocins, which are specialized antimicrobial peptides. The primary mode of action for these bacteriocins involves either pore formation in the target cell membrane or inhibition of cell wall synthesis, ultimately resulting in bacterial cell death (196, 197).

A prominent example is nisin, which exerts its antimicrobial action by binding to Lipid II, a key precursor in bacterial cell wall biosynthesis. This interaction both disrupts peptidoglycan assembly in spore-forming bacilli and promotes the aggregation of nisin–lipid complexes that create pores in the cytoplasmic membrane, leading to loss of membrane integrity and cell death (198, 199).

Certain probiotic strains also demonstrated antiviral capabilities; for instance, *L*. *rhamnosus* GR-1 and *L. fermentum* RC-14 have been shown to rapidly inactivate viral particles such as vesicular stomatitis virus and adenovirus (196). The effectiveness of this antimicrobial activity is well documented across experimental models. *In vitro* studies demonstrated that probiotics such as *Streptococcus salivarius*, *Lactococcus* sp. HY 499, and *Enterococcus faecalis* secrete bacteriocins that inhibit the growth of pathogens, including *Staphylococcus epidermidis*, *Propionibacterium acnes*, and *Staphylococcus aureus* (199–201).

These findings are supported by *in vivo* research, such as the study by Muizzuddin et al. (202), which reported that an extract of *L. plantarum* reduced mild acne lesions, ameliorated erythema, and enhanced skin barrier repair. Other well-documented bacteriocins include bifidocin B from *B*. *bifidum* NCFB, lactacin B from *L*. *acidophilus*, plantaricin from *Lactiplantibacillus plantarum*, and nisin from *L. lactis* (189).

## 6 Dynamics of the human gut microbiota

The establishment of the intestinal microbiota follows a defined sequence of developmental stages. Contemporary evidence suggests that bacterial colonization may begin *in utero*, as indicated by the detection of microorganisms in placental tissue, fetal membranes, amniotic fluid, and umbilical cord blood of healthy mothers and term neonates (203, 204).

This evidence suggests a prenatal initiation of host-microbe interaction. Following birth, microbial colonization of the infant’s gut progresses rapidly, fueled by exposure to a diverse array of sources. These include the maternal vaginal and fecal microbiota, skin, breast milk, and the external environment. The mode of delivery profoundly influences the founding microbial population. Vaginally delivered infants typically develop a gut microbiota that closely mirrors the maternal vaginal flora (205).

In contrast, infants delivered by cesarean section acquire a microbial community dominated by bacteria originating from maternal skin and the oral cavity, as well as microorganisms from the hospital environment and attending healthcare personnel (206). Notably, breast milk serves as a key inoculum of live bacteria, supplying the infant with microbes originating from the mammary ducts, areolar skin, and, significantly, from the maternal gut via an entero-mammary pathway. This process constitutes a crucial natural mechanism for the colonization of the neonatal intestine (206).

The gut microbiota performs essential physiological functions by modulating the expression of genes involved in epithelial immune tolerance, providing vital nutrients to the host, and supporting the maturation and regulation of systemic immune responses (207).

Probiotics are thought to exert beneficial effects through their ability to reshape the composition and function of GI microbial ecosystems positively (208). The human GI tract is estimated to harbor approximately 100 trillion microorganisms, including bacteria, viruses, fungi, and protozoa, collectively encompassing more than 1,000 distinct species (209).

Microbial density remains comparatively low in the stomach and duodenum due to the bactericidal effects of gastric acid, bile, and pancreatic fluid. A steep increase in colonization is observed as it moves distally through the gut, with bacterial loads rising from about 10^4^ cells per gram in the jejunum to 10^9^ cells per gram in the terminal ileum. The colon exhibits the highest microbial density, reaching up to 10¹² anaerobic bacteria per gram of luminal content (210).

The establishment of the body’s normal microbial flora is shaped by localized chemical conditions, oxygen availability, host nutritional status, and the functional state of the immune system. Consequently, the mechanisms by which probiotics exert their effects are intricate and typically strain-specific. The health benefits conferred by probiotics result from a dynamic, multi-faceted relationship between the administered probiotic, the indigenous gut microbiota, and the host (211).

These interactions span multiple domains, including metabolic, microbiological, physiological, neurological, endocrinological, and immunological, often occurring in concert (28). Research indicates that certain probiotic strains can enhance resilience and promote the re-establishment of gut microbial equilibrium following disturbances such as antibiotic therapy or other physiological stressors, even though a precise, universally accepted definition of a “healthy” microbiome remains an ongoing scientific challenge (28).

Wang et al. (209) documented clear disparities in microbial populations between healthy subjects and those with illness or health impairments. Consequently, the composition of an individual’s microbiota is shaped by factors such as age, sex, ethnicity, and geographical region, resulting in a uniquely personalized GI microbial ecosystem (212). This profile is further shaped by genetic predisposition, initial microbial exposure at birth, and long-term dietary habits (209).

Upon entering the intestinal tract, probiotics interact dynamically with the resident bacterial community. The intestinal mucosa serves as the principal defensive interface, functioning as a physical barrier that shields underlying tissues from luminal pathogens and toxins. Probiotics contribute to strengthening the gut’s chemical, biological, immune, and mechanical barrier functions, thereby supporting overall mucosal integrity and host protection (154). Through interactions with intestinal cells, they help normalize permeability, stimulate mucus production, promote regeneration of the mucosal layer, and support the overall integrity and function of the intestinal barrier system (155).

## 7 Probiotic-mediated immunomodulation: mechanisms, regulation, and clinical applications

Probiotics modulate the immune system by secreting immunoregulatory and anti-inflammatory compounds that trigger and fine-tune host immune responses (213). A key mechanism involves stimulating cytokine secretion by immune cells. Cytokines, such as interleukins (ILs), interferons (IFNs), chemokines, tumor necrosis factors (TNFs), and transforming growth factor (TGF), are essential signaling molecules that play a central role in governing innate and adaptive immunity (214, 215).

The immunomodulatory influence of probiotics is further attributed to their direct engagement with a range of immune cells, including IECs, DCs, lymphocytes, and monocytes/macrophages (216). These interactions can be broadly classified as either immunostimulatory, in which specific strains enhance immune activation, or immunoregulatory, in which others help maintain immune homeostasis and promote immunological tolerance (217).

### 7.1 Probiotic-driven immune activation: enhancing host defense

A diverse range of probiotic strains exhibit potent immunostimulatory properties, strengthening host defenses by enhancing pathogen recognition and clearance (218). This activity promotes the production of antibodies, cytokines, and key immune effector cells. Owing to these capabilities, such probiotics are increasingly being explored as adjuncts in immunotherapeutic strategies for specific disease states, including certain forms of cancer (219).

Beyond immune stimulation, certain probiotic strains can finely modulate both the innate and adaptive arms of the immune system, fostering a balanced yet effective immunological response (217).

#### 7.1.1 Reprogramming macrophages: probiotics and phagocytic polarization

As essential components of the innate immune system, macrophages defend the host by phagocytosing and destroying pathogenic microorganisms, including bacteria, viruses, and fungi (220). Probiotics enhance macrophage function by increasing their ability to recognize and eliminate pathogens, thereby strengthening immune surveillance and microbial clearance. A key mechanism underlying this effect is the modulation of macrophage polarization.

Certain probiotic strains promote the pro-inflammatory M1 phenotype, which is particularly effective against intracellular infections, whereas others favor the anti-inflammatory M2 phenotype. The direction of polarization is context-dependent and influenced by cues from the local microenvironment, enabling a tailored immune response that balances pathogen eradication with the resolution of inflammation (221, 222).

Ji et al. (223) reported that *Bacillus amyloliquefaciens* (Ba) modulates the polarization of bone marrow–derived macrophages (BMDMs), promoting both M1 and M2 phenotypes. The study found a particularly strong enhancement of M1 polarization, accompanied by a significant increase in phagocytic activity. This effect was evidenced by improved uptake of soluble antigens, elevated expression of genes encoding IL-6, TNF-α, iNOS, and IL-1β, and a marked increase in nitric oxide production (223, 224).

These results are further supported by *in vitro* studies, which indicate that various probiotic strains can induce nitric oxide release and potentiate the phagocytic function of macrophages (224).

#### 7.1.2 Boosting innate defense: probiotics and natural killer (NK) cell cytotoxicity

NK cells are fundamental elements of the innate immune system, playing a crucial role in defending against tumorigenesis and viral infections (225). They are capable of distinguishing healthy cells from aberrant cells, the latter often exhibiting downregulated or absent major histocompatibility complex (MHC) class I molecules (226). Following target recognition, NK cells can eliminate infected or malignant cells either directly through cytolytic mechanisms or indirectly via the secretion of immune mediators such as TNF-αand interferon-γ (IFN-γ) (194).

The effector functions of NK cells are modulated by DCs, whose activity can, in turn, be influenced by lactic acid bacteria. This tripartite interaction can enhance downstream anti-tumor and anti-viral immune responses. For example, in patients who had undergone surgical resection of colonic polyps, supplementation with *Lactobacillus casei* Shirota (LcS) was associated with a significant reduction in colorectal cancer recurrence (194).

This protective effect is attributed to the ability of *Lactobacillus casei* Shirota (LcS) to stimulate the secretion of TNF-α and interleukin-12 (IL-12), cytokines that are well-established drivers of enhanced NK cell cytotoxic activity (194). Moreover, human DCs primed with lactic acid bacteria can promote both the proliferation and cytotoxic activity of NK cells, resulting in an elevated output of IFN-γ (194). The underlying mechanism involves the potentiation of anti-tumor responses in NK cells by specific *Lactobacillus* strains, a conclusion supported by additional research confirming that LcS augments NK cell activity *in vivo* through an IL-12-dependent pathway (227).

#### 7.1.3 Fine-tuning neutrophil responses: reactive oxygen species (ROS), extracellular traps, and inflammation control

Neutrophils are fundamental innate immune cells that execute a critical phagocytic function, eliminating pathogens through engulfment and destruction (228). Probiotics can precisely modulate neutrophil effector functions by activating specific pathogen-sensing signaling pathways, an effect that is demonstrably strain dependent (229).

The principal mechanisms underlying this modulation include regulation of hydrolytic enzyme activity, generation of ROS during phagocytosis, chemokine-driven recruitment, formation of neutrophil extracellular traps (NETs), and control of inflammatory cytokine secretion. Demonstrating this strain-specificity, *L*. *rhamnosus* GG has been shown to inhibit NET formation, thereby reducing ROS production and limiting the tissue damage associated with chronic inflammatory responses (230).

This ROS suppression is attributed to the strain’s inhibitory effect on the NF-κB signaling pathway. Conversely, in a Sprague-Dawley rat model of sepsis, cell-wall extracts from *Lactobacillus gasseri* ATC33323 enhanced the release of inflammatory mediators, including MIP-1α, MCP-1, TNF-α, and IL-1β (231).

It is essential to emphasize that research into the modulation of neutrophil signaling by probiotics is still in its early stages. Current evidence indicated that the effects on cellular pathways are profoundly influenced by both the specific probiotic strain and the particular immune context under investigation (232).

#### 7.1.4 Probiotics and inflammasomes: balancing inflammation and antiviral defense

Inflammasomes are sophisticated intracellular multiprotein complexes that function as master regulators of innate immunity (233). These multiprotein complexes play a pivotal role in orchestrating the inflammatory response by enabling caspase-1–dependent activation of the potent pro-inflammatory cytokine’s interleukin-1β (IL-1β) and interleukin-18 (IL-18) upon detection of cellular danger signals (233).

Although essential for maintaining intestinal homeostasis, dysregulated inflammasome activity is a key driver in the pathogenesis of chronic IBD. Their activation is triggered by the recognition of pathogen-associated molecular patterns (PAMPs), such as bacterial flagellin, and damage-associated molecular patterns (DAMPs), including uric acid crystals. In addition to mediating antibacterial defense, inflammasomes are integral to antiviral immunity by promoting type I interferon production and initiating pyroptosis, a highly inflammatory form of programmed cell death (234, 235).

Probiotics modulate this system chiefly by influencing interleukin-1β (IL-1β) production and by directly affecting the assembly, activation, and functional capacity of the inflammasome complex. By targeting IL-1β, interleukin-18 (IL-18), and type I interferon pathways, probiotics can fine-tune inflammasome activity, thereby suppressing aberrant inflammatory responses while simultaneously strengthening host antiviral defenses (236).

#### 7.1.5 Modulation of innate immunity and TLRs signaling

The initial interaction involves the recognition of microbe-associated molecular patterns (MAMPs), such as peptidoglycan and lipoteichoic acid, by TLRs expressed on intestinal epithelial cells and DCs (237). Unlike pathogens that trigger an aggressive inflammatory cascade, specific probiotic strains (e.g., *L. rhamnosus* GG and *B. breve*) modulate TLR signaling (specifically TLR2 and TLR4) to activate a controlled immune surveillance state without inducing overt inflammation.

In this regard, Aghamohammad et al. (2023) indicated that this interaction enhances macrophage phagocytic activity and NK cell cytotoxicity, thereby bolstering the host’s first line of defense against viral and bacterial infections (238).

#### 7.1.6 Enhancement of mucosal humoral immunity (IgA)

Probiotics play a pivotal role in reinforcing the mucosal barrier by stimulating B-lymphocytes to differentiate into plasma cells. This process increases the production of SIgA (239). SIgA serves as an “immune exclusion” antibody, binding to pathogens and toxins in the lumen to prevent them from adhering to the epithelium or penetrating the gut barrier (237). Clinical data reviewed by Ebrahimpour-Koujan et al. (240) highlights that supplementation with multi-strain probiotics significantly elevates fecal sIgA levels in pediatric populations, providing a mechanistic explanation for the reduced incidence of respiratory and GI infections.

#### 7.1.7 Postbiotics and metabolite-driven regulation

Emerging research emphasizes that viable bacteria are not always required to elicit an immune response; their metabolic byproducts (postbiotics), particularly SCFAs like butyrate, are potent immunomodulators (241).

Butyrate acts as a HDAC inhibitor in immune cells, suppressing the expression of pro-inflammatory cytokines (TNF-α, IL-6) and enhancing the integrity of epithelial tight junctions. Yang et al. (242) reported that SCFA-producing probiotics not only reduce local gut inflammation but also exert systemic effects via the gut-lung axis, protecting against respiratory inflammation.

### 7.2 Immune balance and tolerance: regulatory roles of probiotics

#### 7.2.1 Cytokine reprogramming: anti-inflammatory and tolerogenic pathways

Acting as essential molecular messengers, cytokines play a central role in orchestrating immune and inflammatory responses and regulate a wide spectrum of additional physiological processes (243). Probiotics largely mediate their beneficial effects by altering patterns of cytokine release from immune cells, thereby promoting a more balanced immune state. This form of immunomodulation is highly strain-specific (**Figure 4**).

Certain probiotic strains direct mucosal immune activity by inducing the secretion of key cytokines and B-cell growth–promoting factors, including interleukin-4 (IL-4), interleukin-5 (IL-5), and interleukin-13 (IL-13) (215, 244). Moreover, probiotics are able to prime the adaptive immune system by enabling a cytokine-facilitated crosstalk, which establishes a complex web of communication between various immune cell lineages (245).

The activation of cytokine synthesis through discrete immunogenic routes is a hallmark feature of numerous probiotic microorganisms (214, 246). Among the most important anti-inflammatory properties of certain probiotics, particularly species of the *Bifidobacterium* and *Lactobacillus* genera, is their ability to upregulate the production of regulatory cytokines such as interleukin-10 (IL-10) and transforming growth factor-β (TGF-β) (165). The biological roles of these cytokines are integral to the tolerogenic and inhibitory actions of regulatory T cells.

In 2025, a recent study of Hu et al. (247) demonstrated that *Bifidobacterium* strains effectively restore immune homeostasis in colitis models by upregulating regulatory T cell populations, which directly correlates with reduced tissue damage. Emerging evidence indicates that certain probiotic strains can potently induce the expansion and enhance the functional activity of regulatory T cells.

For example, a study by Zhao et al. (248) demonstrated that a probiotic consortium comprising *Bifidobacterium*, *Lactobacillus*, and *Enterococcus* species significantly increased the frequency of CD4^+^CD25^+^Foxp3^+^ regulatory T cells within the mesenteric lymph nodes of mice with colitis (248). This expansion was accompanied by an upregulation of the cytokines interleukin-2 (IL-2), interleukin-4 (IL-4), and interleukin-10 (IL-10) in colonic tissues. Concurrently, probiotic treatment induced a marked downregulation of the pro-inflammatory mediators interferon-γ (IFN-γ) and tumor necrosis factor-α (TNF-α) (248).

IL-10 is especially recognized as a preeminent anti-inflammatory and multifunctional cytokine within the immune framework (249). Its mechanism involves acting on antigen-presenting cells (APCs) and directly affecting regulatory T cells to induce the development of T cells that secrete IL-10. The influence of interleukin-10 (IL-10) is extensive, as it shapes immune responses by modulating the biological functions of multiple immune cell populations. For example, IL-10 promotes the rapid expansion, maturation, and survival of B cells while simultaneously stimulating the production of immunoglobulins IgG and IgA (249, 250).

Clinical studies provide evidence to substantiate these mechanisms. In patients with ulcerative colitis, a probiotic regimen consisting of *E. faecalis*, *L*. *acidophilus*, and *Bifidobacterium* was shown to reduce the recurrence rate of flare-ups. This clinical improvement correlated with upregulation of anti-inflammatory IL-10 and concurrent downregulation of the pro-inflammatory cytokines TNF-α and IL-1β (251).

Separately, intranasal administration of *Lactobacillus casei* CRL 431 has been shown to enhance the immune response to *Streptococcus pneumoniae* infection, as evidenced by increased levels of interleukin-10 (IL-10), interleukin-4 (IL-4), IgA, and TNF-αin the respiratory tract (252).

Furthermore, probiotics help maintain immunological equilibrium by attenuating pathological inflammation through the inhibition of pro-inflammatory cytokine signaling and suppression of the nuclear factor-κB (NF-κB) pathway (253). As a master regulator of immune responses, the nuclear factor-κB (NF-κB) transcription factor complex governs the expression of numerous inflammation-related genes, including those encoding pro-inflammatory cytokines such as TNF-α and interleukin-6 (IL-6), as well as chemokines and adhesion molecules. The suppression of NF-κB activation by various probiotics consequently diminishes the production of these mediators, leading to an attenuated inflammatory response (254).

The role of probiotics in immunomodulation is particularly well-documented in ulcerative colitis. Clinical research has shown that an eight-week supplementation with *L. delbrueckii* and *L*. *fermentum* in individuals with moderate ulcerative colitis led to a significant alleviation of intestinal inflammation. This clinical improvement was corroborated by a marked reduction in interleukin-6 (IL-6) concentrations, decreased expression of TNF-α and the NF-κB p65 subunit, and the normalization of other key inflammatory biomarkers (255).

The anti-inflammatory mechanisms are primarily mediated through the inhibition of the NF-κB signaling cascade. For example, strains such as *Lactobacillus casei* and *Lactobacillus paracasei* suppress the production of pro-inflammatory cytokines by inhibiting the phosphorylation and subsequent degradation of IκBα, an endogenous inhibitor of NF-κB. This inhibition prevents the release and nuclear translocation of the NF-κB p65 subunit, thereby attenuating downstream inflammatory signaling (256, 257).

Various other probiotic species employ distinct mechanisms to suppress this pathway. For example, *Lactiplantibacillus plantarum* reduces the DNA-binding capacity of NF-κB, thereby limiting the transcription of pro-inflammatory genes (258), whereas *Lactobacillus brevis* inhibits the phosphorylation of upstream kinases such as IRAK1 and AKT (259). Additional strains, including *Bifidobacterium infantis* and *S. salivarius*, have similarly been shown to attenuate NF-κB activation (254).

#### 7.2.2 Pattern recognition in check: probiotics and TLRs signaling

A pivotal mechanism through which probiotics influence the host immune system and gut microbiota is the regulation of TLRs (237). As a principal class of pattern recognition receptors (PRRs), TLRs are essential for detecting conserved PAMPs originating from a wide range of microorganisms. Activation of TLRs is a cornerstone of innate immunity, triggering key defense responses against invading pathogens (260).

Ligand binding to TLRs typically recruits the adaptor protein myeloid differentiation primary response 88 (MyD88), thereby triggering major pro-inflammatory signaling cascades, including the MAPK and nuclear factor kappa B (NF-κB) pathways (165, 259).

Accumulating evidence indicates that certain probiotic strains can selectively activate these receptors. For example, *Lactobacillus casei* CRL 431 has been shown to activate TLR4 in healthy mice, suggesting a potential role in natural immunological surveillance against bacterial infections (232). Stimulation of TLR4 generally induces the production of pro-inflammatory cytokines, modulates expression of associated receptors (e.g., TLR2), and facilitates immune cell recruitment, culminating in coordinated effector immune responses in systemic organs such as the spleen. Collectively, these TLR-dependent processes are vital for containing and limiting bacterial proliferation (261).

In contrast, excessive or sustained activation of TLRs can promote chronic inflammation and has been implicated in the pathogenesis of numerous immune-mediated disorders (262). Certain probiotics counteract this hyperactivation by downregulating the expression of specific TLRs, thereby limiting excessive immune stimulation and supporting metabolic and immunological balance. For instance, *Clostridium butyricum* TO-A, particularly when co-administered with butyrate, has been shown to lower both the transcriptional and translational levels of TLR4 in HT-29 intestinal epithelial cells (263).

In addition to altering receptor expression, selected probiotics modulate intracellular TLR signaling cascades, thereby suppressing downstream induction of inflammatory mediators. This mechanism contributes to the precise regulation of immune activity and protection against excessive inflammatory responses (263).

These findings are supported by *in vitro* research by Finamore et al. (264), which demonstrated that *Lactobacillus amylovorus* attenuates TLR4-driven inflammatory signaling. Using models of enterotoxigenic *E*. *coli* (ETEC) K88 infection in human Caco-2/TC7 cell lines and porcine intestinal explants, the study showed that *L. amylovorus* reversed ETEC-induced suppression of TLR4 pathway activity. This immunomodulatory effect was mediated through the inhibition of phosphorylation of key NF-κB signaling components, including IKKα, IKKβ, and IκBα (264).

#### 7.2.3 Intracellular sensing: probiotics and nucleotide-binding oligomerization domain-like receptors pathway regulation

NLRs are cytosolic, membrane-associated pattern recognition receptors that play a pivotal role in immune surveillance, particularly within tissues exhibiting low TLR expression (261). Among the more than 20 identified NLRs, NOD1 and NOD2 are the most extensively characterized (261, 265).

Several immunobiotic probiotics, including strains of *L. delbrueckii* subsp., have been shown to upregulate NLR expression. Specifically, *L. bulgaricus* NIAI B6 and *L. gasseri* JCM1131T enhance NLRP3 expression in the gut-associated lymphoid tissue (GALT) of both newborn and adult pigs. These findings suggest that such probiotics may promote NLRP3 activation through integrated signaling pathways involving cross-talk between TLRs and NOD receptors (266).

NLRP3 is also considered a key regulator of intestinal inflammation in humans, as exemplified by conditions such as Crohn’s disease. Dysregulated NLRP3 expression can disrupt immune homeostasis and is associated with various auto-inflammatory disorders (250).

#### 7.2.4 Orchestrating adaptive immunity: probiotics and DCs programming

Probiotics exert significant effects on the development and functional activity of DCs (267). Distinguished by their characteristic branched morphology, these specialized antigen-presenting cells are pivotal for initiating adaptive immune responses through the capture of antigens and their subsequent presentation to T and B lymphocytes (268).

Resident in various sites along the GI tract, DCs are instrumental in determining immune outcomes. They achieve this by regulating the differentiation of naïve CD4^+^ T cells into specific T-helper (Th) subpopulations, such as Th1, Th2, and Th17, via the provision of co-stimulatory signals and the secretion of distinct cytokines (214, 269, 270).

The nature of this immunomodulation is highly strain-specific. For example, *L*. *rhamnosus* has been shown to suppress the rapid proliferation of immature and memory T cells and to reduce the secretion of interleukins-2, -4, and -10 following T-cell receptor activation (165). In contrast, oral administration of the same species *in vivo* enhanced the differentiation of CD4^+^ T cells into both Th1 and Th2 subtypes (271).

These strain-dependent effects are primarily mediated through the engagement of specific pathogen-recognition receptors on DCs, including TLR2, TLR9, and nucleotide-binding oligomerization domain-containing protein 2 (NOD2) (165).

## 8 Encapsulation methods for targeted probiotic delivery

### 8.1 Challenges in preserving probiotic viability

To elicit their beneficial health impacts, probiotics must be administered at a minimum threshold of 10^7^ CFU/mL of live bacteria (272, 273). A significant obstacle to achieving this is the susceptibility of probiotics to a confluence of hostile conditions, including gastric acidity, bile salts, temperature variations, and antimicrobial factors such as hydrogen peroxide, which can severely diminish their viability and functionality (273).

### 8.2 Encapsulation as a protective strategy

To counter these impediments, encapsulation has emerged as a cutting-edge protective strategy. This technique acts as a barrier, protecting microbial cells from degradation. It consequently increases probiotic survival during storage and transit through the upper GI tract, enabling optimal colonization and activity within the colon. The resulting improvement in delivery precision translates directly into greater formulation stability and enhanced therapeutic efficacy (274).

Innovations in nanotechnology have been instrumental in advancing this field, providing a platform for engineering specialized nanocarriers that effectively encapsulate and target the administration of probiotics (275).

### 8.3 Essential criteria for encapsulation matrices

Fundamentally, the process involves enveloping viable organisms in a protective, biocompatible matrix that serves as a shield against deleterious environmental factors. Encapsulating probiotics is a distinctively complex endeavor due to the imperative of maintaining the viability of live microbial cells (276). This necessity imposes strict criteria on the encapsulating polymer, requiring it to be not only biocompatible and biodegradable but also permeable enough to allow for the critical bidirectional exchange of nutrients and metabolites that sustain probiotic life (277).

The success of an encapsulation strategy is fundamentally determined by the selection of the matrix material, which may be derived from natural or synthetic sources. Commonly used natural polymers include alginate, carrageenan, whey protein, gelatin, chitosan, cellulose acetate phthalate, and locust bean gum. Among these, alginate is particularly favored because of its notable thermal stability (60–80 °C) and its proven ability to preserve cell viability under acidic conditions (278). As such, these encapsulation methodologies are indispensable for shielding probiotics and enhancing their survival (279).

### 8.4 Techniques for probiotic encapsulation

While spray drying is an efficient, scalable, and widely applied technique in the food industry, particularly for the biofortification of products such as fruit juices, its application to heat-sensitive probiotics is limited. The high temperatures intrinsic to the process can markedly reduce microbial viability, thereby compromising the functional efficacy of the final product. Co-encapsulation refers to the strategic incorporation of two or more active compounds into a singular, unified carrier system. This approach is often selected over the individual encapsulation of each component due to its enhanced process efficiency and superior cost-effectiveness (280).

Beyond these practical advantages, the technology is also valued for its capacity to markedly enhance the shelf stability and functional preservation of sensitive ingredients, properties that have contributed to its extensive adoption in the pharmaceutical sector. Within this technological domain, the extrusion technique is esteemed as a particularly mild method for immobilizing probiotics (281). It is favored for its straightforward implementation, low operational cost, and its characteristically low shear stress, which collectively promote high cell viability and enable the efficient encapsulation of substantial probiotic populations, most commonly utilizing alginate-based polymers. Conversely, emulsion-based systems, alternatively termed two-phase systems, constitute another fundamental strategy for probiotic encapsulation (281).

Although specific processing steps may vary, the fundamental principle is to generate a stable dispersion of the probiotic culture within a continuous biopolymer matrix, whose composition is precisely engineered to meet the functional and stability requirements of the intended application (281).

### 8.5 Industrial and clinical applications

Probiotic encapsulation technology (PET) has demonstrated considerable promise in both preclinical and clinical studies, leading to its integration into a variety of commercial products. A key challenge, however, is maintaining the long-term viability of the encapsulated cells. In the European market, probiotic ingredients are commonly found in pharmaceutical and nutraceutical products sold in pharmacies and supermarkets (282).

Encapsulation is extensively utilized in nutritional supplements to safeguard probiotic cells during industrial processing and long-term storage, as well as to protect them from the harsh physicochemical conditions encountered in the GI tract. Similarly, the food and beverage industry incorporates encapsulated probiotics into products such as yogurt, cheese, and fermented beverages to extend shelf life and improve stability during rigorous processing (282). In the pharmaceutical field, encapsulation technology enables targeted delivery of probiotics to specific sites in the body, thereby supporting therapeutic interventions for conditions such as GI disorders (283).

### 8.6 Functional properties of encapsulation polymers

Regarding encapsulation matrices, carrageenan, which is FDA, FAO, and WHO-approved compound, serves not only as a feed additive but also as an effective material for probiotic encapsulation. It forms a gel matrix well suited for cell immobilization, setting at room temperature and thereby enhancing probiotic stability during storage and GI transit (284). Due to their amphoteric nature, whey proteins can be effectively combined with carrageenan and pectin. When the pH drops below their isoelectric point, their net charge becomes positive, allowing electrostatic interactions with negatively charged polysaccharides and making them highly appropriate for immobilization applications (285).

In contrast, chitosan—a positively charged polysaccharide obtained by deacetylating chitin, precipitates and becomes insoluble at pH values above 5.4. This property restricts probiotic release in higher-pH environments, thereby limiting its applicability in certain delivery systems. Conversely, cellulose acetate phthalate (CAP) remains insoluble at pH values below 5. Although it does not form beads like chitosan, CAP serves effectively as a coating material, enhancing the overall stability and controlled-release performance of encapsulation systems (286).

### 8.7 Advances in microencapsulation research

Among the established methods for encapsulating microbial cells, extrusion and emulsification are the most widely applied. The extrusion technique is particularly straightforward, amenable to automation, and enables the formation of gelled beads with a high probiotic cell load. Conversely, emulsification creates capsules that may consist of either oil-based or water-based droplets (287).

Microencapsulation functions as a shielding strategy, protecting sensitive compounds from degradation by environmental factors, including oxygen, temperature fluctuations, moisture, enzymes, and gastric acids (288). An additional advantage is the controlled, targeted delivery of the encapsulated core materials to specific sites within the GI tract or other intended locations. Numerous biopolymers are employed as encapsulating matrices for bioactive food ingredients (289).

Contemporary research has increasingly focused on utilizing cereal-derived components to enhance the viability of probiotics in functional foods through microencapsulation. The potential of high-amylose corn starch granules as a probiotic carrier has also been investigated (290).

One study demonstrated that adhering *Bifidobacterium* strains to these granules resulted in a markedly improved survival rate during *in vitro* simulated GI transit (291). Additional research has explored the use of calcium alginate for probiotic microencapsulation in yogurt, where its combination with the prebiotic corn starch synergistically enhanced the encapsulation efficiency and viability of probiotic cells (292).

Furthermore, techniques like spray drying, which yield uniformly coated microcapsules containing live microorganisms, remain a significant and active focus of scientific inquiry in this domain (293).

## 9 Probiotics and human health

### 9.1 Broad therapeutic horizons of probiotics

The therapeutic potential of probiotics has been the subject of extensive research across a broad spectrum of human health conditions (294). Substantial evidence supports the role of probiotics in managing GI disorders, atopic eczema, allergies, and respiratory tract infections. Ongoing research further explores their potential benefits in obesity, metabolic diseases such as type 2 diabetes, cardiovascular health, and cognitive and mental function. Further areas of investigation include the potential roles of probiotics in supporting bone health; managing nonalcoholic fatty liver disease (NAFLD) and hepatic encephalopathy; modulating tumor necrosis factor-α (TNF-α)–mediated inflammation, and contributing to interventions for autism spectrum disorder, burn wound healing, and a range of gynecological conditions (128).

To ensure efficacy, specific administration guidelines have been established. Probiotics are generally most effective when administered on an empty stomach, which facilitates their survival during gastric transit (128). Storage requirements vary according to formulation: heat-dried products typically require refrigeration at 4 °C, whereas lyophilized (freeze-dried) preparations remain stable at ambient temperature. A standard dosage of 1 to 10 billion (10^8^–10^9^) CFUs, administered once or twice daily, is typical. For patients on concurrent antibiotic therapy, it is crucial to separate the probiotic and antibiotic doses by at least two hours to prevent a reduction in probiotic viability (128).

### 9.2 Probiotics in oncological care: promise and limitations

Cancer persists as a predominant cause of global mortality. A considerable number of these deaths are attributable to modifiable risk factors, particularly diet and lifestyle, with dietary practices alone implicated in roughly half of these cases (295). Emerging evidence from *in vitro* and animal models is increasingly highlighting the pivotal role of intestinal and gut microbiota in mitigating the risk of mortality from diet-related causes. Notably, probiotics have shown considerable promise in reducing the incidence of certain cancers, especially colorectal and bladder cancer (296, 297).

Carcinogenesis in the colon has been linked to pathogenic bacteria, such as *Helicobacter, Pseudomonas,* and *Acinetobacter*, a process that can ultimately lead to colorectal cancer. A depletion of beneficial gut flora often facilitates the proliferation of these harmful bacteria. Probiotics mitigate gut dysbiosis by modulating the intestinal microbiome, with frequently investigated strains including *L. acidophilus* and *Lactobacillus casei* Shirota (298, 299).

The mechanisms through which probiotics inhibit the proliferation and progression of colorectal cancer are multifactorial. Their action involves normalizing the intestinal flora and reinforcing the GI barrier. A key beneficial mechanism is the production of SCFAs. These crucial metabolites serve as an energy source for the colonic mucosa, enhance intestinal barrier integrity, and promote the regeneration of colonic epithelium. Furthermore, SCFAs aid in regulating the luminal pH, suppress the proliferation of cancerous cells, and induce apoptosis (300).

Notably, SCFAs act as critical signaling metabolites that activate G-protein–coupled receptors (GPCRs), thereby suppressing pro-inflammatory cytokine production and promoting regulatory T-cell expansion within the colonic mucosa (301). Nevertheless, the precise molecular mechanisms by which probiotics contribute to colorectal cancer prevention and therapy remain insufficiently elucidated (244).

Owing to the pronounced heterogeneity of probiotic strains, each characterized by unique functional attributes and mechanisms of action, their biological effects are inherently complex and frequently strain-specific. Rigorous clinical investigations are warranted to delineate the regulatory networks influenced by probiotics in colorectal cancer, elucidate their sequential mechanisms of action, and facilitate their integration as adjuvant therapeutic modalities for colorectal cancer prevention and management (302).

Promising interventional approaches include the oral administration of live probiotics or purified bioactive metabolites, either as monotherapies or in combination with conventional anticancer agents, to potentially impede colorectal carcinogenesis (303).

Additionally, co-administration of probiotics with standard treatments, such as surgery, chemotherapy, and immunotherapy, may help ameliorate treatment-associated adverse events, augment therapeutic efficacy, and improve overall patient quality of life. Currently, conventional probiotic formulations are already employed in clinical settings as adjunctive therapy for colorectal cancer, largely to minimize surgical complications and alleviate chemotherapy-induced toxicity (304).

### 9.3 Probiotics in burn wound management

The skin serves as the body’s principal physical barrier against microbial invasion; however, any break in this protective layer allows wounds to become susceptible to colonization by pathogens (305). Although Gram-positive bacteria, particularly *S. aureus*, a common constituent of the resident skin microbiota, remain the predominant cause of wound infections, the Gram-negative pathogen *Pseudomonas aeruginosa* is increasingly recognized as an important etiological agent (305).

Consequently, alternative therapeutic approaches, such as probiotic-based interventions, are being actively explored. Probiotic microorganisms support the proliferation of commensal bacteria and have shown efficacy in both the prevention and treatment of cutaneous infections, as demonstrated in clinical studies (306). Topical probiotic formulations, in particular, are emerging as promising strategies in wound care, with their effectiveness largely attributed to the secretion of antimicrobial metabolites, including hydrogen peroxide (306) and bacteriocin (307), which inhibit the growth of pathogens and reduce the risk of infection.

Farahani et al. (308) investigated the integration of probiotics into wound dressings to address challenges in burn care, such as infection and increasing antimicrobial resistance. The investigators employed *in situ*–gelling microparticles encapsulating *Lactiplantibacillus plantarum*, a probiotic strain with well-documented wound-healing potential. Their findings demonstrated that these probiotic-enriched microparticles markedly enhanced the management of burn wound infections. Histological evaluation further showed that the therapeutic efficacy of the probiotic treatment was comparable to that of silver sulfadiazine ointment (308).

Nonetheless, critical challenges related to optimal delivery and long-term stabilization of probiotics persist and warrant further investigation. To ensure protection against both Gram-positive and Gram-negative pathogens, specialized formulations, such as hydrogels (309), films (310), ointments (311), and gels (312), are often required. Encapsulation within natural polymeric matrices such as sodium alginate and chitosan offers a highly promising approach to improving probiotic viability and long-term stability (313).

Moreover, angiogenesis, the formation of new microvasculature from pre-existing blood vessels, constitutes a critical prerequisite for effective wound repair, as it restores essential oxygen and nutrient delivery to the injured tissue. In a paradox of physiology, however, the dysregulation of this very process is a well-established contributor to the pathogenesis of numerous disorders, including cancer and diabetic retinopathy (314).

Notably, the probiotic yeast *S. boulardii* has demonstrated significant efficacy in mitigating inflammatory processes and consequent tissue damage. The yeast elicits these protective effects through mechanisms that reduce visceral hypersensitivity and modulate pro-inflammatory cytokine signaling, thereby establishing a microenvironment that promotes tissue recovery (314).

### 9.4 Probiotics in GI disorders

Probiotics hold considerable promise as therapeutic agents for a spectrum of medical conditions, encompassing lactose intolerance, GI and urogenital infections, ulcerative colitis, neoplasms of the digestive tract, and Crohn’s disease, as shown in **Table 3**. The mechanisms underlying their therapeutic efficacy are multifaceted, encompassing the competitive exclusion of pathogenic microorganisms from mucosal adhesion sites and the biosynthesis of antimicrobial metabolites that inhibit the proliferation of harmful microbes (2).

**Table 3:** Systemic therapeutic potential (oncology, gastroenterology, endocrinology (diabetes), cardiology, and neurology (gut-brain) of probiotics: a multi-disciplinary synthesis of mechanistic pathways and clinical efficacy.

Therapeutic area	Probiotic strain(s)	Disease target	Mechanisms of action	Clinical outcome	References
Oncology	*Lacticaseibacillus rhamnosus* GG	Colorectal cancer	Modulates epithelial turnover; induces mitochondrial apoptosis; enhances antitumor immunity (DC/Tc activation).	Preclinical reduction in tumor burden; preliminary clinical evidence of beneficial mucosal modulation.	(315, 316)
	*Akkermansia muciniphila*	Melanoma	Improves mucosal integrity; reduces metabolic endotoxemia; stimulates anti-tumor T cell responses.	Higher abundance correlates with improved immunotherapy treatment responses in humans.	(97, 317, 318)
	*Lactobacillus casei* Shirota	Bladder cancer	Activates NK cells, increases IFN-γ production, and modulates systemic immune surveillance.	Reduced recurrence risk in non-muscle invasive bladder cancer with long-term consumption.	(319, 320)
	*Saccharomyces boulardii*	Colorectal cancer	Promotes epithelial repair, neutralizes bacterial toxins, and reduces intestinal inflammation.	Reduced inflammation and improved intestinal recovery in preclinical/small human studies.	(321, 322)
	*Clostridium butyricum* and *Faecalibacterium prausnitzii*	Colorectal cancer	Produces butyrate; induces Tregs; modulates epigenetic regulation of tumor cells.	Reduced tumor growth and inflammation in preclinical models.	(323, 324)
	*Lactobacillus acidophilus*	Breast cancer	Induces apoptosis, modulates estrogen metabolism, and regulates immune responses.	Tumor suppression and reduced proliferation were observed in *in vitro* and mouse models.	(325, 326)
Gastroenterology	*Lactobacillus rhamnosus* GG	Acute diarrhea (Pediatric)	Inhibits pathogen adhesion, enhances barrier integrity, and increases SIgA levels.	Reduced duration/severity of acute diarrhea, notably in rotavirus cases.	(327, 328)
	*Saccharomyces boulardii*	AAD and *C. difficile*	Neutralizes toxins (Toxin A/B); promotes epithelial repair; inhibits pathogen overgrowth.	Lower incidence of antibiotic-associated diarrhea (AAD) and reduced *C. difficile* recurrence.	(329, 330)
	*Bifidobacterium infantis* 35624	IBS	Normalizes cytokine balance (↓IL-6/TNF-α, ↑IL-10); modulates visceral hypersensitivity.	Improved bowel habit satisfaction; reduced bloating and abdominal pain.	(85, 331, 332)
	*Lactobacillus reuteri* DSM 17938	Infantile colic	Produces reuterin; reduces inflammation; modulates intestinal motility.	Significant reduction in daily crying time in breastfed infants.	(333, 334)
	*Esherichia coli* Nissle 1917	Ulcerative colitis	Enhances barrier function; induces the expression of antimicrobial peptides; modulates TLR signaling.	Efficacy comparable to mesalazine in maintaining disease remission.	(335, 336)
	*Lactobacillus plantarum* 299v	IBS and functional GI	Adheres to mucosa; competes with pathogens; improves barrier function.	Alleviated abdominal pain and bloating; improved stool consistency.	(337–339)
Endocrinology (Diabetes)	*Lactobacillus plantarum* and *Bifidobacterium breve*	Type 1 diabetes	Modulates gut composition; reduces autoimmune beta-cell destruction; modifies immune tolerance.	Delayed onset in animal models; improved metabolic/inflammatory profiles in humans.	(340–342)
	*Lactobacillus rhamnosus* GG	Gestational diabetes	Modifies maternal metabolism, lowers systemic inflammation, and modulates the microbiota.	Potential reduction in gestational diabetes risk during pregnancy.	(343, 344)
	*Lactobacillus casei* Shirota	Type 2 diabetes	Enhances SCFA production; strengthens barrier; reduces LPS translocation (endotoxemia).	Improved insulin sensitivity; decreased fasting plasma glucose and cytokines.	(345, 346)
	*Bifidobacterium lactis* Bb12	Type 2 diabetes	Modulates microbiota/barrier; improves glucose and lipid metabolism.	Decreased fasting blood glucose and HbA1c (adjunct to lifestyle changes).	(347–349)
	*Lactobacillus reuteri* DSM 17938	Type 2 diabetes	Increases GLP-1 secretion; improves insulin sensitivity; lowers oxidative stress.	Enhanced insulin sensitivity and reduced inflammatory markers.	(347, 350)
	*Bifidobacterium animalis* subsp. *lactis* 420	Type 2 diabetes and obesity	Strengthens barrier; reduces endotoxemia; modulates energy metabolism.	Decreased weight gain; enhanced insulin sensitivity; beneficial microbiota shifts.	(351, 352)
Cardiology	*Lactobacillus reuteri* NCIMB 30242	Hypercholesterolemia	High bile salt hydrolase (BSH) activity increases cholesterol catabolism and reduces cholesterol absorption.	Reduced LDL-C (9–12%), total cholesterol, and apoB in RCTs.	(353–355)
	*Lactobacillus plantarum* 299v/GLP3	Dyslipidemia	Produces SCFAs; modulates bile signaling (FXR/TGR5); reduces TMAO formation.	Modest LDL-C reduction; decreased plasma TMAO in high-risk patients.	(356)
	*Lactobacillus helveticus*	Hypertension	Produces ACE-inhibitory tripeptides; reduces angiotensin II; improves endothelial NO.	Small reductions in SBP/DBP in mild hypertension (results heterogeneous).	(357, 358)
	*Bifidobacterium longum* BB536/CCFM1077	Hyperlipidemia	BSH activity: remodels bile acid pools; reduces TMAO and inflammation.	Decreased total/LDL cholesterol; significant TMAO reduction in unstable angina.	(356, 359)
	*Akkermansia muciniphila*	Atherosclerosis risk	Strengthens barrier; reduces endotoxemia (LPS/TLR4); modulates GLP-1/AMPK.	Improved metabolic/inflammatory profiles; reduced atherosclerotic lesions (preclinical).	(360–362)
Neurology (Gut-Brain)	*Lactobacillus rhamnosus* JB-1	Anxiety or depression	Modulates GABA receptors, lowers corticosterone, and regulates vagus nerve signaling.	Improved mood/stress resilience; reduced anxiety-like behaviors (preclinical).	(363–365)
	*Bifidobacterium longum* 1714	Stress/anxiety	Regulates HPA axis; lowers cortisol; modulates neurotransmitter pathways.	Enhanced sleep quality, stress resilience, and cognitive function under stress.	(366, 367)
	*Lactobacillus helveticus* R0052, and *Bifidobacterium longum* R0175	Depression/anxiety	Modulates GABA/serotonin; reduces systemic inflammation; strengthens barrier.	Significant reduction in HADS scores.	(368, 369)
	*Lactobacillus plantarum* PS128	ASD and Parkinson’s	Regulates neuroinflammation; increases the levels of dopamine/serotonin precursors.	Improved motor scores (Parkinson’s) and social behaviors (ASD).	(370, 371)
	*Bifidobacterium breve* A-1	Alzheimer’s or cognition	Upregulates BDNF; suppresses neuroinflammation; modulates immune response.	Improved MMSE scores; reduced inflammatory cytokines in elderly adults.	(372–374)
	*Clostridium butyricum* MIYAIRI 588	Stroke and Parkinson’s	Increases butyrate; enhances neuroplasticity; reduces systemic inflammation.	Improved motor function (Parkinson’s); enhanced motor recovery post-stroke.	(375, 376)

AAD, antibiotic-associated diarrhea; ACE, angiotensin-converting enzyme; AMPK, AMP-activated protein kinase; ASD, autism spectrum disorder; BDNF, brain-derived neurotrophic factor; BSH, bile salt hydrolase; DC, dendritic cell; GABA, gamma-aminobutyric acid; GI, gastrointestinal; GLP-1, glucagon-likepeptide-1; HADS, hospital anxiety and depression scale; HbA1c, glycated hemoglobin; HPA, hypothalamic-pituitary-adrenal; IBS, irritable bowel syndrome; *C. difficile, Clostridioides difficile*; IFN, interferon; IgA, immunoglobulin A (sIgA, secretory immunoglobulin A); IL, interleukin; LDL-C, low-density lipoprotein cholesterol; LPS, lipopolysaccharides; MMSE, mini-mental state examination; NK, natural killer; NO, nitric oxide; SCFA, short-chain fatty acid; TLR, toll-like receptor; TMAO, trimethylamine N-oxide; TNF-a, tumor necrosis factor alpha; Tregs, regulatory T cells; FXR, farnesoid X receptor; SBP, systolic blood pressure; DBP, diastolic blood pressure; RCTs, randomized controlled trials.

Particular strains confer distinct clinical benefits; notably, *Lactiplantibacillus plantarum* demonstrates utility in mitigating manifestations such as distension and visceral pain, and *S. boulardii* is applied therapeutically to control diarrhea and augment intestinal function (377). A substantial and expanding body of scientific evidence highlights the significant therapeutic potential of probiotics in the management of diverse diarrheal disorders. Their prophylactic efficacy in preventing traveler’s diarrhea is particularly well established (378, 379), alongside a well-defined beneficial role in managing acute diarrhea in children (380).

The strains *S. boulardii* and *L*. *rhamnosus* GG are among the most widely researched and are frequently associated with successful clinical outcomes for these indications. Contrasting these positive findings, an extensive Cochrane meta-analysis of 82 studies concluded that the ability of probiotics to abbreviate the duration of acute infectious diarrhea, while detectable, is modest and remains surrounded by significant uncertainty (381).

A more persuasive and consistent body of evidence exists for the use of probiotics to prevent antibiotic-associated diarrhea. Both *S. boulardii* and *L. rhamnosus* GG have proven effective in diminishing the incidence of antibiotic-associated diarrhea (382–384) and, with particular relevance, in preventing diarrhea associated with *C*. *difficile* infection (385, 386).

A critical insight from this research is the strong correlation between higher dosage regimens and increased prophylactic efficacy. Probiotic research also encompasses IBD, where strains such as *S. boulardii*, *L*. *rhamnosus* GG, and other lactobacilli, bifidobacteria, and *S. thermophilus* have been employed as adjunctive therapeutic agents. However, their capacity to sustain remission in IBD patients is not conclusively established (387, 388). Although several meta-analyses indicated that probiotics can contribute to the induction of remission (387–391), the supporting data are more compelling for ulcerative colitis than for Crohn’s disease (389, 390).

Specific multi-strain formulations also exhibit promise in both preventing the initial onset of pouchitis and in maintaining remission following its successful induction. Concerning IBS, including its constipation-predominant variant (IBS-C), the aggregate benefit derived from probiotic supplementation is typically marginal (392). Most clinical trials to date have employed strains from the *Lactobacillus* and *Bifidobacterium* genera. Nevertheless, a meta-analysis of 15 randomized controlled trials (RCTs) demonstrated that probiotic supplementation significantly shortened whole-gut transit time, improved stool consistency, and increased stool frequency (392).

Probiotic supplementation has produced heterogeneous, yet overall favorable, results in ameliorating the symptoms of lactose intolerance (393, 394). Moreover, a compelling body of evidence endorses the use of specific probiotic strains as an adjunct to standard triple therapy, enhancing the eradication rates of *H. pylori* in patients with peptic ulcer disease (395).

This is corroborated by the research of Wilkins and Sequoia (396), whose study involving 1,163 children and adults demonstrated that *Lactobacillus*-based probiotics significantly improved eradication rates when used concomitantly with antibiotics compared to a placebo control. Within neonatology, although the methodological rigor of available studies is frequently limited (397, 398), emerging evidence suggests that specific probiotic strains may confer protection against necrotizing enterocolitis in preterm infants. The therapeutic purview of probiotics also extends to hepatology, with data indicating that they can ameliorate parameters of NAFLD (399) and are effective in managing hepatic encephalopathy.

A meta-analysis of six RCTs encompassing 496 adults with cirrhosis demonstrated that probiotic therapy significantly reduced the risk of progression to overt hepatic encephalopathy (400). Strains with high bile salt hydrolase activity and a documented influence on bile acid pools, such as *Lactobacillus casei*, *B. longum*, and *A. muciniphila*, are natural candidates for the nodes relating to farnesoid X receptor (FXR)/TGR5 signaling, improved lipid handling, and reduced hepatic steatosis (401, 402).

**Figure 5:**
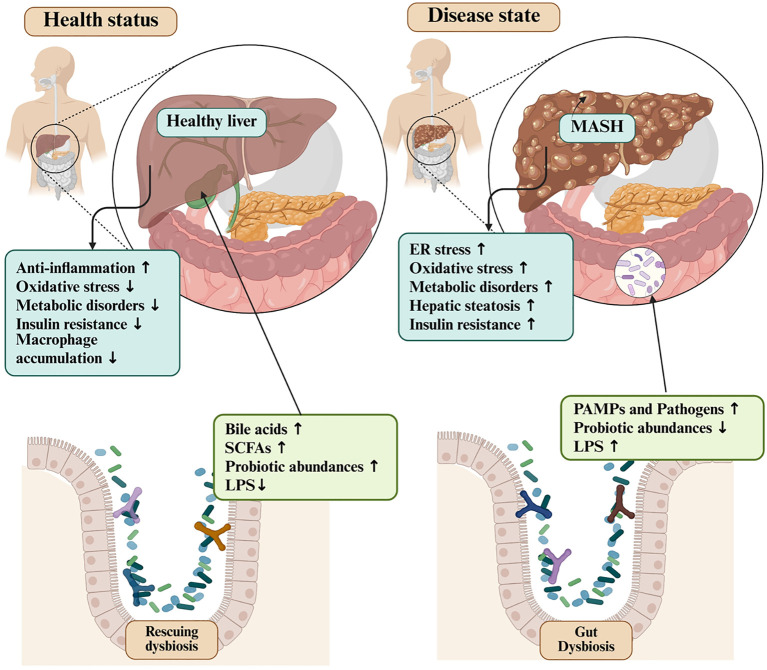
The impact of dysbiosis on hepatic metabolic and inflammatory status via the gut-liver axis. MASH, metabolic dysfunction-associated steatohepatitis; ER, endoplasmic reticulum; PAMPs, pathogen-associated molecular patterns; LPS, lipopolysaccharides; SCFA, short-chain fatty acid.

**Figure 6:**
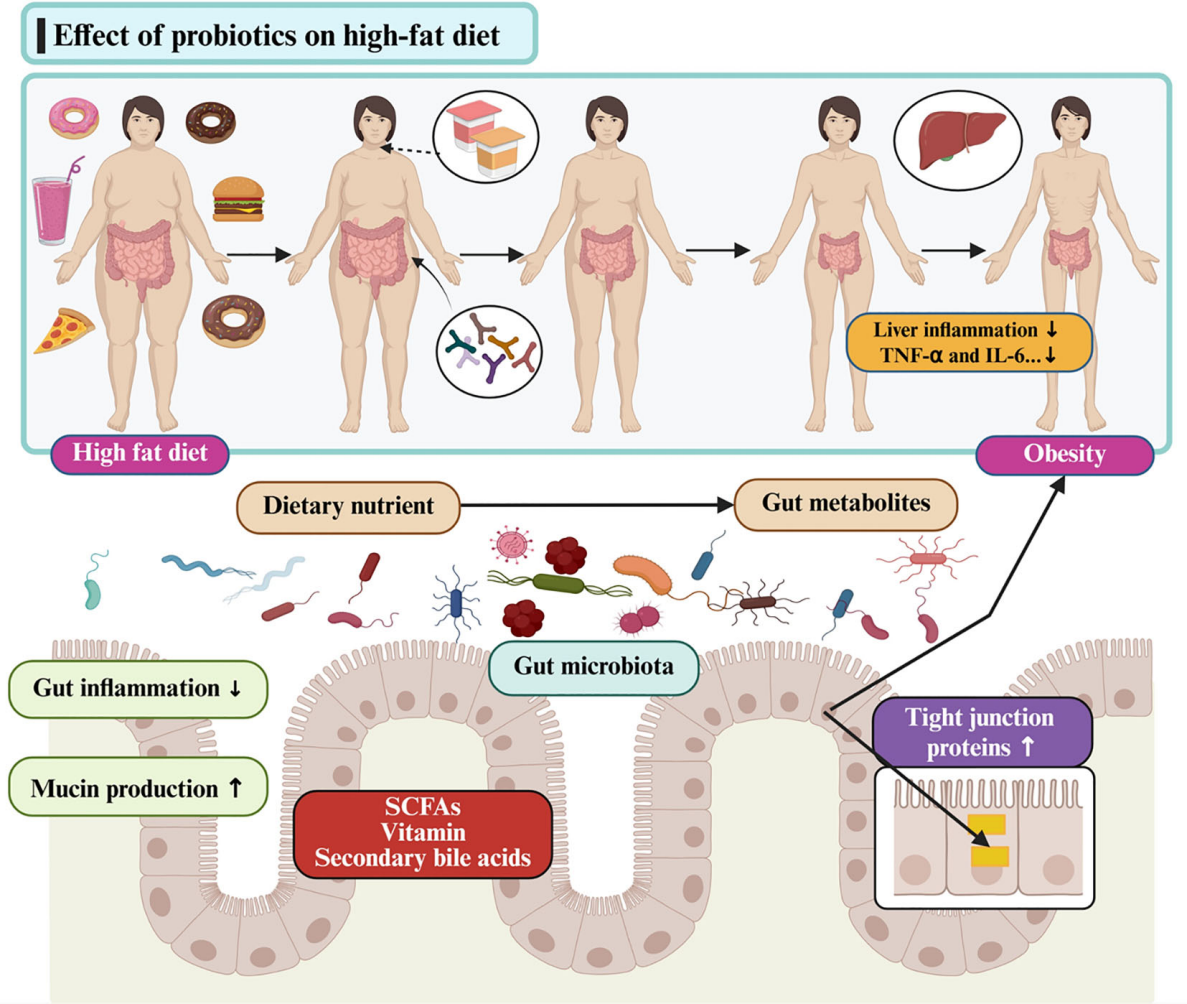
Impact of probiotic administration on mitigating high-fat diet-induced obesity and metabolic inflammation via the gut-liver axis. TNF-a, tumor necrosis factor alpha; IL-6, Interleukin-6; SCFA, short-chain fatty acid.

**Figures 5** and **6** focus on hepatoprotection and the gut–liver axis.

### 9.5 Dermatological applications of probiotics

Recent studies examined the administration of probiotics during pregnancy, lactation, and/or early infancy (403–406). These studies exclusively examined the efficacy of specific probiotic strains in reducing the risk of atopic eczema in children. The results demonstrated that only certain strains of *Lactobacillus* spp., *Bifidobacterium* spp., and *Propionibacterium* spp. were beneficial, while other strains within the same species showed no effect (405).

Beyond atopic eczema, probiotics have also been investigated as adjunctive therapies for acne. *In vitro* studies have shown that strains such as *Lactobacillus salivarius* LS03, along with *Lactococcus* and *S. salivarius*, produce bacteriocins capable of inhibiting the growth of *Cutibacterium acnes* (407).

Similarly, *Bifidobacterium adolescentis* SPM0308 has demonstrated antimicrobial activity that suppresses the proliferation of both *C. acnes* and *S. aureus* (408). Moreover, *S. thermophilus* has been shown to enhance the skin’s lipid barrier by stimulating ceramide synthesis in the stratum corneum, thereby improving skin hydration, and by generating phytosphingosine, which further exerts inhibitory effects against *C. acnes* (407).

In the treatment of acne vulgaris, a study by Espinoza-Monje et al. (409) used the strain *Weissella viridescens* UCO-SMC3. Both topical and oral administration led to a favorable modulation of the inflammatory response, with a significant reduction in acne lesions reported among participants after cream application (409).

Notably, Espinoza-Monje et al. (409) were the first to characterize the antimicrobial and immunomodulatory properties of this strain, which was originally isolated from garden slime (*Helix aspersa* Müller). The therapeutic potential of probiotics extends to other dermatological conditions as well. Clinical studies have reported positive effects of oral probiotics on psoriasis, including significant improvements in patient outcomes (410).

Supporting these findings, a 12-week RCT by Navarro-López et al. (411) involving 90 patients found that probiotic supplementation significantly reduced the Physician Global Assessment (PGA) index. Additionally, a reduced risk of relapse was observed six months after the intervention ended (411).

### 9.6 Probiotics in obesity and cardiometabolic health

Among the multiple factors driving the modern escalation of obesity, the most prominent are an inherited genetic susceptibility and a sustained energy imbalance, defined by a chronic excess of caloric intake relative to energy expenditure (412). Adipocyte-derived hormones, notably leptin and adiponectin, are integral to the development of obesity. Research indicates that the probiotic strain *Lactobacillus gasseri* BNR17 can ameliorate weight gain by inhibiting these hormonal pathways (413).

Moreover, certain probiotics aid in weight management by stimulating the adrenergic nervous system to trigger thermogenesis. Specific strains, including *L. acidophilus*, *Lactobacillus casei*, and *B. longum*, are shown in **Table 3**. The mechanism of these strains has demonstrated hypocholesterolemic effects through their ability to reduce serum levels of triglycerides, low-density lipoprotein (LDL), and high-density lipoprotein (HDL) (414).

Beyond modulating lipid profiles, some probiotics also assist in regulating blood pressure (415). This is achieved via bioactive peptides synthesized by strains such as *Lactobacillus helveticus* and *S. cerevisiae*; these peptides act as inhibitors of the angiotensin-converting enzyme (ACE), a key regulator of hypertension (416).

Accumulating evidence indicates that alterations in gut microbiota composition can compromise its functional efficacy and disrupt intestinal barrier integrity, precipitating a state of low-grade chronic inflammation. This persistent pro-inflammatory milieu plays a critical role in promoting adiposity and fostering insulin resistance in the host, as shown in **Table 3**. Consequently, the gut microbiota has gained recognition as a key regulator of appetite and an important determinant in the pathogenesis of obesity (417).

Current research suggests that probiotic supplementation may facilitate weight management and improve cardiometabolic parameters (418). Systematic reviews document modest yet consistent reductions in body weight, waist circumference, and visceral adipose tissue among overweight and obese individuals following probiotic administration, with *Lactobacillus* species representing the most extensively investigated strains (419–422).

Another meta-analysis revealed that specific microbial strains, including *B. breve*, *B. longum*, *S. thermophilus*, *L. acidophilus*, *L. delbrueckii*, and *Lactobacillus casei*, confer beneficial effects on both anthropometric measures and metabolic risk factors, such as fasting glucose and insulin levels, in populations with metabolic disorders (423).

Proposed mechanisms underpinning these benefits include the facilitation of carbohydrate digestion and absorption, alongside the probiotic-mediated augmentation of SCFA production within the GI tract (424). Furthermore, probiotic interventions appear to ameliorate inflammatory biomarkers (e.g., C-reactive protein), enhance glycemic control, and improve insulin metabolism in patients with type 2 diabetes or metabolic syndrome (425–429), as well as in pregnant women diagnosed with gestational diabetes mellitus (425, 426).

Beneficial effects have likewise been documented with respect to cardiovascular risk factors, including hypertension (430) and dyslipidemia (431, 432). Despite these encouraging findings, substantial heterogeneity across published studies precludes definitive conclusions regarding the therapeutic efficacy of probiotics in obesity and cardiometabolic management. These observations highlight the need for large-scale, multicenter, randomized, placebo-controlled trials to ascertain whether targeted modulation of specific probiotic strains or other gut microbes associated with a lean phenotype can effectively counteract obesity and improve insulin sensitivity.

### 9.7 Probiotics in diabetes management

Accumulating evidence suggests that probiotics exert a modulatory effect on gut hormone secretion, a process crucial for maintaining metabolic homeostasis. By alleviating insulin resistance, a key pathophysiological hallmark of type 2 diabetes, this modulation contributes to improved metabolic homeostasis (433).

The integration of probiotics into the management of type 2 diabetes mellitus (T2DM) has emerged as a promising adjunctive therapy. Recent investigations (2023–2025) have moved beyond simple association to elucidating precise molecular mechanisms, particularly involving the gut-pancreas axis, incretin hormone regulation, and the mitigation of systemic oxidative stress (434).

Moreover, certain probiotic strains have been shown to inhibit adipocyte hyperplasia, suggesting a protective role against the onset of diverse metabolic disorders (**Figure 6**).

#### 9.7.1 Enhancement of incretin signaling glucagon-like peptide-1 and insulin sensitivity

A primary mechanism by which probiotics exert glycemic control is the stimulation of glucagon-like peptide (GLP-1) secretion from enteroendocrine L-cells (435). Probiotic strains, particularly *Bifidobacterium animalis* subsp. *lactis* and *A. muciniphila*, ferment dietary fibers to produce SCFAs such as butyrate and propionate (436). These metabolites activate G-protein coupled receptors (GPR43/41) on intestinal L-cells, triggering the release of GLP-1 (437).

This hormone enhances glucose-dependent insulin secretion, inhibits glucagon release, and slows gastric emptying. A 2025 study involving *Bifidobacterium* strains demonstrated that probiotic-driven tryptophan metabolism produces indole derivatives that activate aryl hydrocarbon receptors (AhR), further upregulating proglucagon gene expression and GLP-1 synthesis (438). Additionally, multi-strain formulations have been shown to significantly reduce insulin resistance (HOMA-IR) by modulating the TGR5 bile acid receptor pathway (399).

#### 9.7.2 *A. muciniphila*: A next-generation metabolic regulator

The mucin-degrading bacterium *A. muciniphila* has garnered significant attention as a therapeutic target for metabolic syndrome (439). It strengthens the intestinal mucosal barrier, preventing the translocation of lipopolysaccharides (LPS) into systemic circulation (metabolic endotoxemia), a key driver of insulin resistance (440).

A quantitative meta-analysis of animal and early clinical studies (2024) indicated that *A. muciniphila* supplementation significantly reduced fasting blood glucose (-21.2%) and improved glucose tolerance (+22.1%) by reinforcing the gut barrier and reducing pro-inflammatory cytokines like TNF-α (441). Further 2025 reviews highlight its safety and efficacy in improving insulin sensitivity in overweight, insulin-resistant cohorts (439).

#### 9.7.3 Oxidative stress mitigation and lipid profile optimization

Chronic hyperglycemia in diabetes induces mitochondrial dysfunction and excessive ROS production. Probiotics function as “bio-antioxidants.” (442). Strains such as *Lactobacillus plantarum* and *Lactobacillus casei* produce antioxidant enzymes (Superoxide dismutase (SOD), glutathione peroxidase - GSH-Px) and exopolysaccharides that scavenge free radicals (442).

A 2025 systematic review confirmed that probiotic supplementation in T2DM patients leads to a significant elevation in total antioxidant capacity (TAC) and a reduction in malondialdehyde (MDA), a marker of lipid peroxidation (442, 443). This antioxidant effect correlated with improved lipid profiles, specifically reductions in triglycerides and LDL-cholesterol.

#### 9.7.4 Clinical outcomes: glycated hemoglobin (HbA1c) and fasting glucose

Meta-analyses of RCTs published between 2023 and 2024 have solidified the clinical relevance of these mechanisms (444). The aggregated data indicated that multi-strain probiotic interventions (typically >8 weeks in duration) result in a statistically significant reduction in fasting blood glucose (FBG) and a modest but meaningful reduction in glycated hemoglobin (HbA1c), particularly in patients with baseline HbA1c > 8.0%. However, the efficacy is strain-dependent, with *Lactobacillus* and *Bifidobacterium* blends showing superior results compared to single-strain interventions (444).

### 9.8 Probiotics in urinary tract infections

Urinary tract infections often originate from an imbalance in the vaginal microbial ecosystem, representing a significant health concern for women across all age cohorts (445). These conditions are medically stratified by location within the urinary system and often recur. Although prophylactic antibiotic therapy can provide temporary protection, it is counterbalanced by the risk of accelerating antimicrobial resistance. The body’s natural defense depends heavily on lactic acid production by symbiotic microorganisms; in particular, endogenous lactic acid bacteria are critical for maintaining the acidic vaginal milieu that deters pathogenic colonization and growth. Among current preventive and therapeutic strategies, probiotic interventions have gained considerable recognition as a promising option (445).

Clinical studies have confirmed the efficacy of more than fifty specialized formulations—primarily comprising *Lactobacillus* strains such as *L. brevis*, *L. reuteri*, *L. vaginalis*, and *L. rhamnosus*—for both the management and prevention of urinary tract infections (446).

### 9.9 Probiotics and the gut–brain axis

Recent investigations position the administration of *Lactobacillus plantarum* as a potentially significant intervention for children with autism spectrum disorder (ASD) (447). This approach is integrated into a broader therapeutic framework in which specific probiotic strains, most notably *Lactobacillus helveticus*, *Lactobacillus casei*, and *L. rhamnosus*, have been associated with alleviating psychological distress, reducing anxiety symptoms, and improving behaviors characteristic of ASD, respectively. The foundational mechanism underpinning these effects is the production of neuroactive metabolites by gut bacteria, which bear structural and functional resemblance to host-derived neurochemicals (448).

Through the gut–brain axis—a complex bidirectional communication network—these probiotics, together with prebiotics, can enhance central nervous system (CNS) function and help alleviate various mental health disorders, including depression, anxiety, ASD, schizophrenia, and Alzheimer’s disease, as shown in **Table 3**. The complex interplay between the gut microbiome, the enteric nervous system, and the brain is facilitated by direct neural connections and indirect endocrine and immunological pathways (449).

Consequently, there is growing enthusiasm for employing prebiotics, probiotics, and synbiotics as innovative, natural adjuncts for managing neuropsychiatric conditions, largely due to their comparatively favorable side-effect profiles compared with traditional drug therapies. Contemporary scientific reviews substantiate their beneficial influence on symptoms of anxiety, depression, chronic stress, sleep disturbances, and cognitive decline associated with Alzheimer’s disease (450).

Research in animal models, particularly rodents, has shown that prebiotic and probiotic interventions can reshape the composition and functional activity of the gut microbiota, thereby modulating neurochemical signaling and attenuating neuroinflammatory processes within the brain (451). Moreover, studies employing germ-free models and probiotic supplementation provide compelling evidence for a causative relationship between microbial metabolites and cerebral health (452).

Notwithstanding these encouraging preclinical results, the current body of evidence is not yet robust enough to formally endorse these interventions for complex disorders such as schizophrenia and ASD. While probiotic supplementation has demonstrated modest efficacy in attenuating depressive symptoms, translating these findings into clinical practice requires more rigorous verification. There is an urgent need for rigorously designed, large-scale, randomized, placebo-controlled trials that evaluate diverse probiotic formulations within well-defined patient cohorts to generate definitive and reproducible outcomes (280, 453).

**Figure 7:**
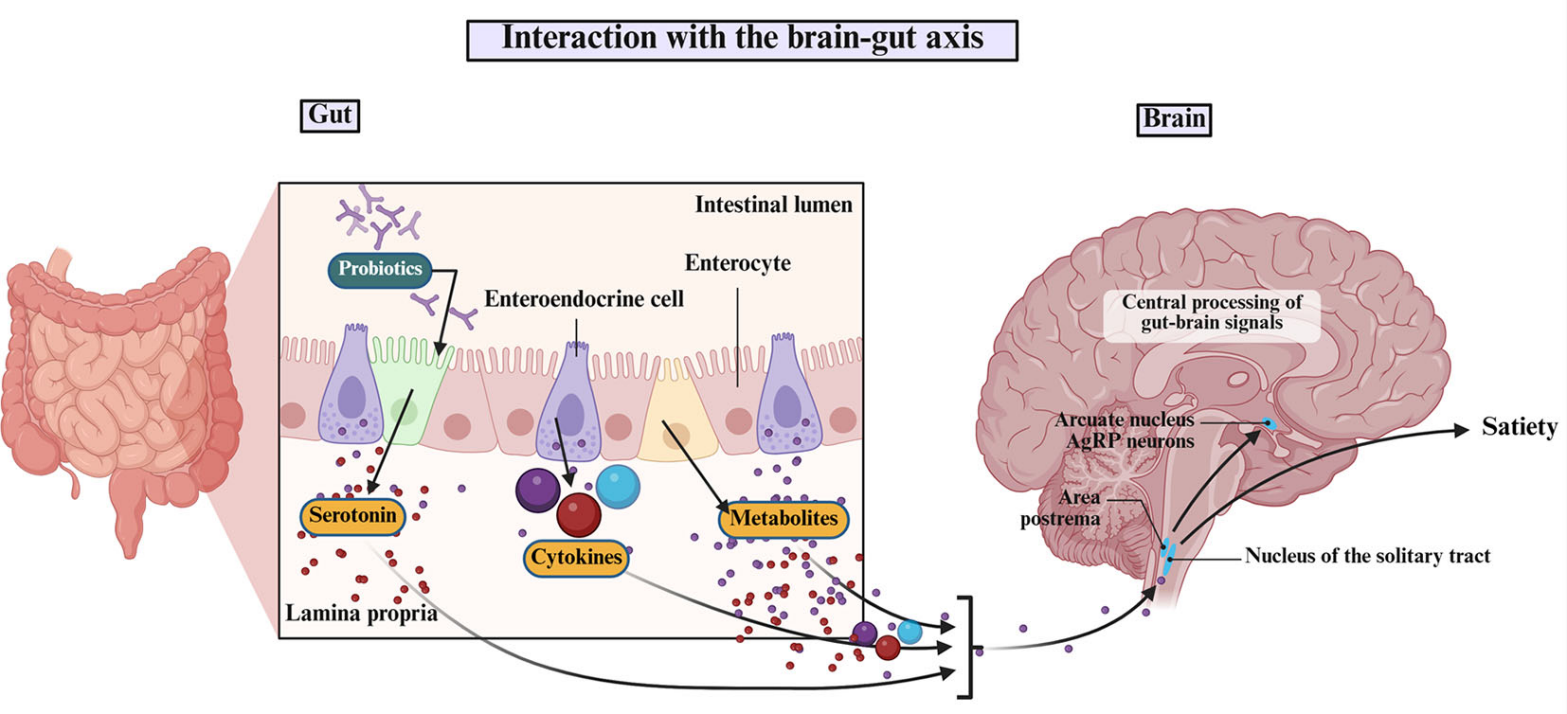
Probiotic-mediated regulation of satiety via the gut-brain axis signaling pathways. AgRP neurons, Agouti-related peptide neurons.

Future research must also prioritize elucidating the precise molecular and biochemical mechanisms by which these therapeutics mediate their effects. **Figure 7** illustrates the gut–brain axis, including vagal pathways, microbial metabolites, and the HPA axis.

### 9.10 Probiotics in inflammatory diseases

Probiotics are under investigation for treating ulcerative colitis and Crohn’s Disease, which are collectively termed IBD (454). IBD is marked by chronic inflammation of the GI tract and is associated with dysbiosis—an imbalance between the gut’s aerobic and anaerobic bacterial communities. Research indicates that specific probiotic strains, including *Lactobacillus*, *Bifidobacterium*, and *Enterobacter*, may help ameliorate this inflammation (455). The mechanism of probiotics as anti-inflammatory agents are detailed in section 7 and **Table 4**, demonstrates their anti-inflammatory efficacy.

**Table 4:** Immunomodulatory and antiviral efficacy of probiotics: Mechanistic pathways targeting systemic inflammation and viral pathogenesis.

Probiotic strain(s)	Disease/Condition Target	Mechanisms of action	Clinical/Experimental outcome	References
Anti-Inflammatory				
*Faecalibacterium prausnitzii*	IBD (Crohn’s or Colitis)	Produces anti-inflammatory MAM protein and butyrate; inhibits NF-κB/IL-12; increases IL-10.	Reduced Crohn’s relapse risk (associated with abundance); decreased colitis severity in animal models.	(456, 457)
*Akkermansia muciniphila*	NAFLD or metabolic inflammation	Modulates endotoxin-induced TLR4 signaling; activates AMPK via outer membrane proteins; strengthens mucin barrier.	Enhanced insulin sensitivity; reduced liver inflammation in NAFLD.	(458, 459)
*Propionibacterium freudenreichii*	Ulcerative colitis	Produces conjugated linoleic acid and propionate; induces apoptosis in pro-inflammatory cells; releases IL-10.	Improved mucosal healing; reduced colitis severity in mouse models.	(460, 461)
*Clostridium butyricum* (MIYAIRI 588)	Colitis, or antibiotic dysbiosis	Inhibits NF-κB activation; stimulates Tregs; produces butyrate (SCFA).	Restored microbiota post-antibiotics; decreased intestinal inflammation in ulcerative colitis patients.	(462, 463)
*Escherichia coli* Nissle 1917	Ulcerative colitis	Induces defensins; competes with pathogens; modulates TLR signaling pathways.	Effective for ulcerative colitis maintenance; efficacy comparable to mesalazine.	(464, 465)
*Streptococcus thermophilus*	Mucosal inflammation	Reduces IL-8 secretion; generates anti-inflammatory exopolysaccharides.	Lowered inflammatory cytokines; enhanced gut barrier function in colitis models.	(466, 467)
*Bacillus coagulans*	IBS and Arthritis	Produces lactic acid; suppresses TNF-α; increases anti-inflammatory IL-10 and TGF-β.	Reduced pain and inflammatory markers in patients with IBS and rheumatoid arthritis.	(466, 467)
Antiviral				
*Lactobacillus rhamnosus* GG, *Saccharomyces boulardii*, and *Bifidobacterium breve*	Viral gastroenteritis	Increases mucosal IgA; modulates DC/Th1 responses; reinforces tight junctions; competes for viral receptors.	Reduced diarrhea duration (up to 1 day) and viral shedding in children.	(70, 468)
*Lactobacillus rhamnosus* GG, *Lactobacillus gasseri*, and *Bifidobacterium animalis*	Influenza	Stimulates TLR-3/7/9 and IFN-I/III; increases NK cytotoxicity; balances Th1/Th2 cytokines.	Lower incidence/duration of respiratory infections; reduced viral loads and antibiotic use.	(72, 469, 470)
*Lactobacillus reuteri* Protectis	Coxsackievirus A16, Enterovirus 71	Binds virions directly; produces reuterin/organic acids to inhibit viral entry and replication.	Inhibited viral replication (*in vitro*); improved survival in animal models.	(471, 472)
*Enterococcus faecium*	Influenza A	Traps viral particles adsorptively; induces IFN-β; enhances macrophage activity.	Suppressed replication (*in vitro*); improved viral clearance in swine models.	(473, 474)
*Lactobacillus paracasei, Lactobacillus plantarum*, and *Lactobacillus reuteri*	HSV-2, vesicular stomatitis	Secretes antiviral metabolites (H_2_O_2_, bacteriocins); activates RIG-I/MAVS pathway and IFN-1 production.	Reduced epithelial viral replication; decreased herpes simplex virus 2 recurrence (murine models).	(475)
*Bifidobacterium infantis* MCC12, and *Bifidobacterium breve* MCC1274	Rotavirus, or influenza	Produces SCFAs to enhance barrier; activates NLRP3-MAVS-IFN axis; modulates gut–lung axis.	Protection against rotavirus (infants); reduced influenza loads and increased antiviral cytokines.	(476, 477)
*Saccharomyces boulardii*	Acute viral diarrhea	Restores microbiota balance; produces proteases to degrade toxins; enhances epithelial repair.	Shortened acute diarrhea duration; recommended by ESPGHAN guidelines.	(478, 479)

IBD, inflammatory bowel disease; IBS, irritable bowel syndrome; NAFLD, non-alcoholic fatty liver disease; ESPGHAN, European Society for Paediatric, Gastroenterology, Hepatology and Nutrition; NF-kB, nuclear factor kappa B; IL, interleukin; TLR, toll-like receptor; AMPK, AMP-activated protein kinase; Tregs, regulatory T cells; SCFA, short-chain fatty acid; TNF-a, tumor necrosis factor alpha; TGF-b, transforming growth factor beta; IgA, immunoglobulin A; Th, T helper cell (Th1/Th2); DC, dendritic cell; IFN, interferon; NK, natural killer; RIG-I, retinoic acid-inducible gene I; MAVS, mitochondrial antiviral-signaling protein; NLRP3, NLR family pyrin domain containing 3.

### 9.11 Antiviral applications of probiotics

A critical appraisal of the evidence supports probiotic interventions against a range of clinically significant viral infections, including SARS-CoV-2, viral upper respiratory tract infections (URTIs), influenza, viral hepatitis, human immunodeficiency virus (HIV), and human papillomavirus (HPV) (480, 481). It further examines the potential of probiotics as adjunctive therapies to enhance immunogenicity and improve overall vaccine efficacy. **Table 4** illustrates the antiviral effectiveness of probiotics.

#### 9.11.1 Probiotics and SARS-CoV-2 infection

Probiotics possess considerable therapeutic potential in the management of respiratory infections, primarily through their capacity to modulate immune responses and attenuate inflammation (482). This rationale spurred extensive investigation of diverse probiotic strains during the SARS-CoV-2 pandemic. Numerous clinical trials, primarily utilizing immunomodulatory *Lactobacillus* and *Bifidobacterium* strains, were subsequently undertaken to assess their efficacy against COVID-19 (483).

The collective results from these trials suggest a strong association between probiotic supplementation and marked symptomatic improvement. The most reliably documented advantages encompass a reduction in fatigue and the amelioration of GI disturbances.

Rathi et al. (484) reported that a synergistic formulation of probiotics (*B. coagulans*, *B. subtilis*, and *Bacillus clausii*) combined with digestive enzymes significantly alleviated physical and mental fatigue; after 14 days, 91% of treated patients showed improvement, compared with only 15% in the placebo group. In a separate double-blind RCT, a formulation containing *Lactiplantibacillus plantarum* strains KABP022, KABP023, and KABP033, supplemented with *Pediococcus*, accelerated recovery and significantly reduced the overall duration of symptoms compared with a placebo (485).

Further corroborating the benefits for digestive health, an additional RCT noted that COVID-19 patients administered probiotics displayed more considerable improvement in GI symptoms and a decreased rate of hospital-acquired diarrhea (486). Complementing these clinical findings, analyses of the gut microbiota have identified distinct microbial signatures associated with COVID-19.

Harper et al. (480) reported that an increase in opportunistic pathogens, including *Actinomyces*, *Erysipelaclostridium*, *Streptococcus*, *Veillonella*, *Rothia*, and *Enterobacter*, was positively correlated with both the initial diagnosis and the severity of the illness. In contrast, higher abundances of beneficial butyrate-producing bacteria, such as *Faecalibacterium*, *Anaerostipes*, and *Bifidobacterium*, were inversely associated with both the occurrence and severity of the disease (480).

#### 9.11.2 Probiotics for viral upper respiratory tract infections

The upper respiratory tract, including the nostrils, nasal and oral cavities, tonsils, pharynx, and larynx, is susceptible to a range of infections collectively termed upper respiratory tract infections (URTIs). These encompass the common cold (primarily affecting the nasal mucosa), as well as tonsillitis, pharyngitis, laryngitis, acute otitis media, and sinusitis (487).

Major viral pathogens associated with these infections comprise rhinoviruses, respiratory syncytial viruses, adenoviruses, influenza and parainfluenza viruses, coronaviruses, and human metapneumovirus (487). Zhao et al. (488) reported that extensive clinical investigations have explored the role of probiotics in preventing URTIs. The proposed protective mechanisms are thought to parallel those implicated in GI infection prophylaxis, primarily by modulating immune function at both local and systemic levels (488).

Key immunomodulatory actions include enhanced phagocytic activity of peripheral leukocytes, upregulated secretion of immunoglobulins (IgA, IgG, and IgM), and increased production of cytokines such as interleukins, TNF-α, and interferon-α (IFN-α) (488). Supporting these findings, a meta-analysis by Poon et al. (489) focusing on *Lactobacillus paracasei* subsp. *Paracasei* CNCM I-1518 confirmed that probiotic supplementation offers significant potential for the prevention of URTIs.

#### 9.11.3 Probiotics in influenza infection management

Human influenza A, B, and C viruses exhibit pronounced tropism for respiratory epithelial cells, initiating infection by attaching and replicating in the respiratory mucosa (490, 491). Although most influenza infections are self-limiting and resolve without intervention, specific demographic and clinical factors heighten susceptibility to severe manifestations. Notably, individuals at the extremes of age, both infants and the elderly, as well as those with pre-existing comorbidities, particularly cardiopulmonary disorders, are at markedly increased risk (490). Compelling evidence from animal models substantiates the therapeutic potential of probiotics.

Belkacem et al. (492) demonstrated in a murine influenza model that administration of *Lactobacillus paracasei* CNCM I-1518 conferred substantial protection, evidenced by reduced host susceptibility, attenuated pulmonary inflammatory cell infiltration, and accelerated viral clearance. Further rodent studies emphasized the gut microbiota’s integral role in orchestrating systemic immune responses, indicating that a balanced microbiome conditions the lung milieu for defense by modulating interferon (IFN) signaling pathways. This preemptive antiviral state limits initial viral replication, thereby attenuating early disease progression (493).

Wu et al. (494) demonstrated the adverse immunological consequences of microbial disruption, showing that antibiotic-induced dysbiosis in rodents suppressed the expression of key innate immune mediators, including TLR7 and NF-κB mRNA. As a result, antiviral immunity was significantly compromised, a deficiency that was reversed following supplementation with probiotics from the *Bifidobacterium* and *Lactobacillus* (sensu lato) genera (494).

#### 9.11.4 Adjunctive probiotic therapy in viral hepatitis

Hepatitis B (HBV) and hepatitis C (HCV) infections constitute a major worldwide public health burden, with a disproportionately high impact on populations in developing regions (495). The underlying pathogenesis reflects a complex interplay between viral activity and the host immune response, driving progressive liver injury that can ultimately lead to serious complications such as cirrhosis and hepatocellular carcinoma (495).

Lee et al. (496) reported that a cellular extract of *Bifidobacterium adolescentis* SPM0212 exhibited potent anti-HBV activity in an *in vitro* model employing HepG2.2.15 cells—a hepatocyte-derived line stably transfected with HBV DNA and constitutively secreting hepatitis B surface antigen (HBsAg). The study postulated that the mechanism involves potentiation of the Mx GTPase pathway, a critical mediator of interferon-induced antiviral defense (496).

Conversely, other research (497) has documented a marked elevation in *S. salivarius* levels concurrent with the progression of chronic hepatitis C (CHC). Further analyses have revealed pronounced gut microbial dysbiosis in cirrhotic patients with hepatic encephalopathy (HE), marked by increased abundances of bacterial families such as Alcaligenaceae, Porphyromonadaceae, and Veillonellaceae, as well as genera including *Enterococcus*, *Megasphaera*, and *Burkholderia* (498).

These compositional changes are implicated in the pathogenesis of hyperammonemia and exacerbation of systemic inflammatory processes. Notably, a prospective RCT provided evidence that probiotic intervention contributes effectively to the primary prevention of hepatic encephalopathy in cirrhotic individuals (499). The protective effects of probiotics are largely attributed to their capacity to suppress urease-producing, pro-inflammatory pathogenic bacteria, thereby reducing the risk of HE development (499).

#### 9.11.5 Probiotic interventions in HIV infection

A defining feature of HIV infection is the profound and rapid depletion of CD4^+^ T lymphocytes (500), a loss that intensifies with advancing disease (501, 502). The administration of probiotics has gained traction as a potential adjunctive treatment in HIV management, owing to their ability to foster immune tolerogenesis (503), displace pathogenic bacteria through competitive exclusion, and attenuate inflammatory processes (504).

These mechanisms are thought to mitigate T-cell loss and senescence, thereby supporting more robust immune reconstitution once viral suppression is achieved. In an HIV-positive cohort, Blázquez-Bondia et al. (505) found that a synbiotic formulation comprising two strains of *Lactiplantibacillus plantarum* and *Pediococcus acidilactici*, along with prebiotic fibers, was safe and well tolerated.

The role of vaginal microbiota is also critically important in the context of HIV. Specific probiotic strains, including bifidobacteria and *L. rhamnosus*, promote the increased synthesis of SCFAs and vitamins, exert influence on mucosal immunity and epithelial function, reinforce the gut barrier, assist in the prevention of bacterial vaginosis, and lower associated morbidity (506).

A separate clinical trial investigating whether a 30-day probiotic supplementation (1 × 10^9^ CFU/day) could reduce the incidence of diarrhea in HIV patients concluded that extending the treatment duration beyond one month may be necessary to elicit measurable clinical benefits (507). Consequently, although probiotic supplementation is often incorporated into dietary recommendations for these individuals, its application requires careful clinical evaluation (508).

#### 9.11.6 Probiotics and HPV

Recent findings indicated that probiotic-based interventions hold considerable promise for treating HPV infection, facilitating viral clearance and improving cervical cytology (509). In a prospective study of 54 women diagnosed with HPV and low-grade squamous intraepithelial lesions (LSIL), daily probiotic supplementation over six months led to viral clearance in 29% of the treatment group, a rate significantly higher than the 19% observed in controls (510). Further supporting this, a double-blind, RCT involving 121 women with high-risk HPV (HR-HPV) assessed a three-month intravaginal regimen containing *L. rhamnosus* GR-1 and *Limosilactobacillus reuteri* RC-14 (511).

While this specific treatment did not significantly improve genital HR-HPV clearance rates, it was linked to a notable reduction in the incidence of abnormal and unsatisfactory Pap smears (511). Collectively, these findings suggest that extended courses of vaginal probiotic therapy may be more effective than shorter regimens in promoting HPV clearance and resolving cytological abnormalities (512).

Thus, the principal benefit of probiotics in HPV management likely resides in their capacity to restore and reinforce a protective vaginal microbiota, an effect that may outweigh specific treatment variables such as duration or route of administration (480).

#### 9.11.7 Probiotics as vaccine adjuvants

Vaccination confers immunity by pre-sensitizing the adaptive immune system to particular pathogens, thereby eliciting a rapid and robust defensive reaction upon re-exposure (480). Animal model studies have demonstrated that certain probiotic strains can enhance vaccine-induced immune responses and reduce susceptibility to subsequent infections. Consistently, an increasing number of well-designed human studies indicated that adjuvant supplementation with specific probiotics can improve the efficacy of vaccines against influenza, cholera, and various childhood diseases (99).

These observations are supported by a systematic review of clinical trials, which found that probiotics can positively modulate vaccine-induced immune responses (513). The review found that certain probiotics enhance the immune response to influenza vaccination. This benefit is especially significant in older adult populations, who generally demonstrate a weaker antibody response (seroconversion) to such immunizations (514).

A clinical trial conducted by Yeh et al. (515) that administered probiotics as an adjunct to influenza vaccination evaluated outcomes using hemagglutination inhibition (HI) antibody assays. A subsequent meta-analysis reported significant increases in HI titers—20% for A/H1N1, 19.5% for A/H3N2, and 13.6% for influenza B—among participants receiving probiotics compared with control groups. These findings highlight the potential role of the gut microbiome in enhancing the host’s antiviral immune competence.

Furthermore, an independent meta-analysis concluded that probiotic supplementation effectively augments the immunogenic response to influenza vaccination in adults (516).

## 10 Guidance for selecting an optimal probiotic product

### 10.1 Clinical indication as the central criterion

The identification of an optimal probiotic for therapeutic applications remains a dynamic and nuanced process, continually refined by emerging clinical research and trial data. Current scientific consensus emphasizes that the primary criterion for probiotic selection is the specific clinical indication—whether prophylactic, therapeutic, or adjunctive—given the marked differences in efficacy exhibited by individual bacterial strains across distinct health objectives (517).

### 10.2 Patient population and individual needs

The suitability of probiotics depends on factors such as age, physiology, and health status. Pediatric patients, adults, and immunocompromised individuals often require distinct formulations and dosing strategies. Tailoring probiotic therapy to the target population ensures both safety and clinical relevance (517).

### 10.3 Single-strain versus multi-strain formulations

An equally critical consideration is the target patient population, as the suitability of a probiotic is often contingent on factors such as age, with distinct formulations recommended for pediatric versus adult use. Ultimately, the choice between a single-strain or multi-strain product must be guided by a rigorous synthesis of robust, documented clinical evidence rather than anecdotal claims (517).

### 10.4 Quality standards and product transparency

Practical determinants, including regional product availability and the governing regulatory landscape, also significantly influence the selection process. High-quality probiotic supplements are characterized by transparent labeling that explicitly states the manufacturer’s information, the precise identity of all strains included, their quantified potency in CFUs, and the recommended daily dosage (517). Compliance with national regulations governing health and nutritional claims is essential; products lacking this critical information should be approached with caution. Furthermore, in jurisdictions where certain probiotics are regulated as prescription medications, consultation with a physician or a clinically trained pharmacist is imperative (517).

### 10.5 Evidence-based clinical guidelines based on American Gastroenterological Association (AGA) recommendations

For clinicians managing infectious and GI disorders, the AGA provides detailed, condition-specific recommendations (518). In *C. difficile* infection, the administration of probiotics is advised only within the context of a clinical trial.

For the prevention of *C. difficile* infection in adults and children receiving antibiotics, the AGA favors the use of specifically recommended probiotic preparations over no intervention or unspecified alternatives; these include *S. boulardii*, the dual-strain combination of *L. acidophilus* CL1285 and *Lactobacillus casei* LBC80R, and a three-strain blend of *L. acidophilus*, *L. delbrueckii* subsp. *bulgaricus*, and *B. bifidum*, or a four-strain combination of *L. acidophilus*, *L. delbrueckii* subsp. *bulgaricus*, *B. bifidum*, and *S. salivarius* subsp. *thermophilus*. In Crohn’s disease, ulcerative colitis, and IBS, probiotic use for symptomatic children and adults is recommended solely within clinical trial settings (518).

For pouchitis in adults and children, an eight-strain formulation, comprising *L. paracasei* subsp. *paracasei*, *L. plantarum*, *L. acidophilus*, *L. delbrueckii* subsp. *bulgaricus*, *B. longum* subsp. *longum*, *B. breve*, *B. longum* subsp. *infantis*, and *S. salivarius* subsp. *thermophilus*, is recommended over no treatment or alternative regimens. By contrast, for acute infectious gastroenteritis in children, the AGA advises against the use of probiotics (518).

Finally, to prevent necrotizing enterocolitis (NEC) in preterm, low-birth-weight infants, the administration of specific probiotics is strongly endorsed. Recommended options include various *Lactobacillus* and *Bifidobacterium* combinations (e.g., *L. rhamnosus* ATCC 53103 with *B. longum* subsp. *infantis*; *Lactobacillus casei* with *B. breve*; or a specified six-strain product), as well as *B. animalis* subsp. *lactis* (including strain DSM 15954), *L. reuteri* (DSM 17938 or ATCC 55730), and *L. rhamnosus* (ATCC 53103, ATC A07FA, or LCR 35) (518).

### 10.6 International standards and safety frameworks

The foundational international framework for assessing the safety and efficacy of probiotics was established through collaborative efforts by the FAO of the United Nations and the WHO. Preliminary expert recommendations published in 2001 were subsequently formalized into comprehensive guidelines in 2002, which continue to provide pivotal scientific direction (519, 520).

These seminal documents outline standardized protocols for probiotic evaluation, providing overarching principles while addressing specific concerns such as pathogenicity, intrinsic toxicity, and potential allergenicity, as well as assessing functional and nutritional properties (519, 520). This concerted global initiative serves as the cornerstone for assuring the quality and safety of probiotic products utilized worldwide.

Later, the International Scientific Association for Probiotics and Prebiotics (ISAPP) consensus reinforced that “probiotic” should be reserved for strains that are taxonomically defined, safe for their intended use, supported by at least one positive human clinical trial, and present alive at an efficacious dose throughout shelf life (18). Recent ISAPP-oriented quick guides and professional tools seek to translate these criteria into clinical and regulatory practice for healthcare professionals and industry (521, 522).

Global regulatory frameworks now apply the FAO/WHO principles differently across product categories such as foods, food supplements, and live biotherapeutic products (LBPs) (523). In the European Union, the Food Safety Authority (EFSA) relies on the Qualified Presumption of Safety (QPS) approach for microorganisms in foods and has issued strict guidance on health claims, which, in practice, has made approval of “probiotic” health claims very difficult. In contrast, the United States regulates probiotics under multiple pathways (foods, dietary supplements, drugs, biologics), using Generally Recognized As Safe (GRAS) notices for food uses and more demanding drug/biologic pathways for LBPs intended to treat or prevent disease (521).

Recent studies emphasized that regulatory frameworks for probiotics and other “biotics” are advancing rapidly, especially for LBPs, for which conventional drug regulations often do not align well with live microbial products that possess complex, multifactorial mechanisms of action (524). Increasing acknowledgment exists that varying regional regulations—such as those in the European Union, United States, and Asia-Pacific—pose challenges to global market access, generate heterogeneity in permitted terminology (including the use of the term “probiotic”), and hinder the dissemination of consistent, evidence-based information to healthcare professionals and consumers (513).

Notwithstanding the FAO/WHO framework, substantial scientific and regulatory challenges persist (142). Gaps in safety assessment encompass limited long-term data in vulnerable populations, an incomplete understanding of horizontal gene transfer related to antimicrobial resistance, and uncertainty regarding the systemic impact of prolonged microbiome modulation, particularly with next-generation strains lacking a long history of safe use (142).

Furthermore, there remains an incomplete consensus on the optimal preclinical models and safety endpoints for live microbes utilized outside the GI tract or in high-risk clinical environments (525). From a regulatory perspective, inconsistencies across jurisdictions regarding the classification of probiotics, the evidentiary requirements for claims, and manufacturing and quality control standards contribute to variability in product quality and the potential for misleading labeling (526).

The advent of personalized and engineered probiotics introduces further complexity, as factors such as individual microbiome variability, host genetics, and polypharmacy can influence both the efficacy and safety profiles, thereby complicating standardized evaluation (527, 528). Future development of probiotic regulation is expected to move toward more harmonized global standards that still allow flexibility for innovation (523).

Experts increasingly advocate aligning safety expectations for high-risk probiotic products (e.g., LBPs) with those applied to other complex biologicals, including systematic genomic characterization, standardized adverse-event reporting, and structured post-market surveillance, while avoiding unnecessarily stricter requirements than for comparable biologic therapies (525). At the same time, regulatory and scientific bodies are calling for clearer, internationally agreed criteria for the use of the term “probiotic” and for substantiation of health claims, to enhance transparency and consumer trust (526).

On the scientific front, integration of multi-omics, systems biology, and personalized medicine is anticipated to refine strain selection, elucidate mechanisms of action, and support precision probiotic interventions tailored to host microbiome profiles (529, 530). Future frameworks will likely need to incorporate guidance for engineered strains, synbiotics, microbial consortia, and microbiome-based therapeutics, extending the FAO/WHO principles to more complex products while maintaining their core focus on strain specificity, safety, and evidence-based health benefits (531).

## 11 Recent advances and emerging trends in probiotic science

### 11.1 Expanding therapeutic horizons of probiotics

Probiotics constitute a cornerstone in sustaining the homeostasis of the human intestinal microbiota, with a robust body of scientific literature corroborating their extensive health-promoting effects. These beneficial microorganisms demonstrate considerable therapeutic promise in the management of a diverse spectrum of disorders (532).

These applications span acute diarrheal diseases, IBS, a variety of bacterial and viral infections (including HIV and HPV), obesity, insulin resistance syndrome, type 2 diabetes, NAFLD, cancer, allergic disorders, neurological conditions, and lactose intolerance (342, 343). Moreover, probiotics play an essential role in supporting optimal immune function and maintaining a stable gut microbiota (5, 533–536).

### 11.2 Designer probiotics and synthetic biology innovations

While the application of probiotics to address health issues has a long history, contemporary breakthroughs in culturomics—notably through gnotobiotic animal models—have catalyzed the evolution of a novel scientific frontier dedicated to engineering host-specific probiotic therapeutics (537).

A key innovation in this field is the development of “designer probiotics”—genetically engineered or otherwise modified commensal bacterial strains specifically tailored to withstand production stresses such as lyophilization, thermal fluctuations, and gastric acidity, or to enhance their most beneficial functions for the host (537). Through advances in synthetic biology, researchers can reprogram probiotic and commensal bacteria to perform entirely new physiological functions (537).

### 11.3 Fecal microbiota transplantation (FMT): beyond the gut

Another transformative innovation in microbiome-based medicine is FMT, also referred to as bacteriotherapy or stool transplantation (537). This procedure operates on the principle of reconstituting a healthy microbial equilibrium in a recipient’s intestine by transferring processed fecal material from a rigorously screened healthy donor (537).

The infusion of this filtrate into the GI tract serves to counteract dysbiosis by directly replenishing a consortium of beneficial bacteria. Emerging research indicates that FMT may also hold therapeutic value for certain inflammatory and infectious dermatological conditions, such as psoriatic arthritis, psoriasis, and alopecia universalis (533).

### 11.4 Personalized nutrition, nutrigenomics, and nutrigenetics

Given the substantial inter-individual variability in gut microbiota composition, future nutritional strategies are expected to increasingly emphasize dietary personalization tailored to an individual’s unique microbial profile. In this context, the emerging disciplines of nutrigenomics and nutrigenetics are poised to play pivotal roles in developing precise, individualized dietary recommendations. At the same time, the field is advancing with the introduction of NGPs (538).

### 11.5 NGPs: beyond *Lactobacillus* and *Bifidobacterium*

NGPs expand the probiotic idea beyond traditional lactic acid bacteria by focusing on key commensals that are deeply embedded in host–microbiome networks. Unlike classic probiotics, mainly from *Lactobacillus* and *Bifidobacterium* genera, NGPs are usually chosen from groups such as *Bacteroides, Akkermansia*, *Faecalibacterium*, *Eubacterium*, and related butyrate- or propionate-producing species (532, 538).

These microbes hold central roles in food webs and are strongly linked to metabolic and immune balance (29). Whole-genome and comparative metagenomic studies showed that these organisms have detailed pathways for breaking down complex carbohydrates, producing SCFAs, transforming bile acids, and creating surface or secreted molecules that modulate the immune system (95). These features highlight their potential as carefully selected live biotherapeutics, rather than just randomly chosen fermenters.

A key distinction of NGPs lies in their capacity to reshape host–microbiome interactions at multiple levels. Many NGP candidates are significant contributors to butyrate and propionate pools, thereby supplying energy substrates for colonocytes, enhancing epithelial barrier function, and exerting epigenetic and anti-inflammatory effects through HDAC inhibition and G-protein–coupled receptor signaling (96).

Species such as *F. prausnitzii* and *Roseburia* spp. are strongly associated with reduced intestinal inflammation and improved metabolic profiles. Meanwhile, *A. muciniphila* resides within the mucus layer, modulates mucin turnover, and influences bile acid, lipid, and glucose metabolism, positioning it at the interface of barrier integrity and systemic metabolic regulation (44, 97).

Furthermore, some NGPs release extracellular vesicles and other bioactive metabolites that communicate with immune, epithelial, hepatic, and neural tissues, suggesting that their benefits may arise as much from postbiotic signaling as from mere colonization or competitive exclusion (539).

However, translating NGPs into safe, scalable products faces significant regulatory and technological hurdles. Many of these taxa are strict anaerobes with demanding nutritional needs, low tolerance to oxygen, and limited stability outside their native gut environment. This requires specialized fermentation, encapsulation, and cold-chain logistics, which greatly increase production costs compared to traditional probiotics. From a regulatory viewpoint, most NGPs lack a history of safe use and therefore do not qualify for existing “generic” safety designations, such as GRAS or QPS (402).

They need comprehensive toxicology testing, detailed genomic risk assessments for virulence and antimicrobial resistance genes, and rigorous phase I–III clinical trials, similar to other LBPs (525). There is also ongoing debate about the most suitable product category—whether food, supplement, or drug—and about how to standardize measures of potency, viability, and functional outputs for strains whose benefits may vary significantly depending on context, including baseline microbiome, diet, and host genetics.

*A. muciniphila* exemplifies both the promise and the complexity of NGPs (532). Multiple preclinical and early clinical studies associated this species with improved insulin sensitivity, reduced adiposity, lower systemic inflammation, and enhanced gut barrier function in obesity, type 2 diabetes, and related cardiometabolic disorders (532).

Pasteurized preparations as well as derived outer-membrane proteins have demonstrated bioactivity, broadening the scope to postbiotic formats. Concurrently, its localization in the mucus layer raises inquiries regarding long-term effects on mucosal integrity in various host contexts (44).

Additionally, large-scale manufacturing necessitates strict anaerobic processes and meticulous control of batch-to-batch consistency (402). These considerations exemplify a broader theme for NGPs: achieving their therapeutic potential will require integrating high-resolution host–microbiome science with appropriate regulatory frameworks, advanced manufacturing technologies, and precision medicine approaches that align specific NGPs or consortia with defined clinical indications and patient subgroups.

### 11.6 Diversification of probiotic sources and delivery vehicles

Regarding delivery matrices, the future sources of probiotics are expected to diversify beyond traditional fermented dairy, cereals, fruits, and vegetables to encompass novel vehicles such as meat, fish, honey, and even environmental origins like soil (536). Regardless of source, every probiotic strain incorporated into food or supplement formulations must be supported by clear evidence of safety and efficacy.

### 11.7 Ensuring safety and efficacy in novel applications

Rigorous scientific evaluation is essential to exclude virulence factors, pathogenic potential, and the production of harmful metabolites, while also confirming a documented record of safe human consumption (536).

### 11.8 The road ahead: toward precision microbiome-based therapies

Advances in biotechnology and bioinformatics are expected to deepen our understanding of probiotic mechanisms of action, accelerate the discovery of novel candidate organisms and prebiotic substrates, and enhance the reproducibility and comparability of both laboratory and clinical studies (536). Collectively, these innovations—reinforced by large-scale epidemiological investigations—will pave the way for more sophisticated and personalized dietary and clinical applications of probiotics, precisely aligned with an individual’s unique biological and microbial profile. This shift toward personalized microbiome-targeted interventions represents the envisioned future trajectory of probiotic science and therapeutic development (536).

## 12 Potential adverse effects and risk considerations of probiotic supplementation

### 12.1 Historical safety record and regulatory frameworks

For centuries, the consumption of fermented foods containing live microorganisms has been associated with human health benefits, providing a strong historical foundation for their safety. Consistent with this longstanding record, probiotic formulations marketed as dietary supplements are regulated under food safety frameworks rather than the more stringent standards applied to pharmaceuticals (99). These supplements, which are commonly available in powders, capsules, or liquids, are generally considered safe for regular consumption by the general population (99).

### 12.2 Common and typically benign adverse effects

Despite a well-documented spectrum of health benefits, a minority of users may encounter transient, mild adverse effects during an initial period of microbial adaptation (540). The predominant complaints are GI, manifesting as bloating, flatulence, diarrhea, constipation, and nausea (541, 542). These symptoms, especially bloating and gas, typically diminish without intervention within several weeks (542).

Initiation of probiotic supplementation, particularly formulations containing yeast strains, may occasionally cause transient increased thirst during the first week of use, a response that typically resolves spontaneously as the body acclimates (540). Certain probiotic-rich fermented foods, such as sauerkraut and kimchi, contain appreciable levels of biogenic amines (e.g., histamine and tyramine), which, in susceptible individuals, can trigger headaches or migraines. Although some yogurts also contain these amines, their levels are generally much lower. Cutaneous reactions, such as rashes or pruritus, are uncommon and typically related to hypersensitivity to a specific supplement component, with symptoms usually resolving promptly after discontinuation (540).

### 12.3 Rare but serious risks in vulnerable populations

Beyond these common and typically benign reactions, a more serious, albeit uncommon, risk exists that live probiotic organisms could translocate and cause systemic infections, necessitating antimicrobial treatment (99). This risk is significantly elevated in immunocompromised individuals or those with specific comorbid conditions. It is also noteworthy that many contemporary probiotic strains are genetically engineered in laboratory settings to enhance their beneficial properties. Consequently, the safety of each distinct strain must be rigorously evaluated and subject to ongoing surveillance. These safety assessments must unequivocally demonstrate that genetically modified organisms cannot persist in the environment, lack transferable antibiotic resistance markers, and are incapable of disseminating any deleterious genetic material to other microbiota (99).

In conclusion, although the safety profile of probiotics is well established for the healthy majority, caution is warranted in certain vulnerable populations. These include recipients of stem cell or solid-organ transplants receiving anti-rejection therapy; individuals undergoing immunosuppressive treatment such as corticosteroids or chemotherapy, as well as patients with autoimmune disorders; those with structural cardiac abnormalities, including valvulopathies, prosthetic heart valves, or a history of infective endocarditis; and patients with acute abdominal conditions, active IBD such as colitis, neutropenia (either present or anticipated following chemo- or radiotherapy), or active intestinal perforation and leakage (543).

## 13 Safety considerations and potential risks of probiotic use

### 13.1 The need for rigorous strain characterization

Although probiotics are widely regarded as safe for the general population, their extensive use across diverse demographic groups, including infants, the elderly, and individuals with compromised immune function, necessitates rigorous and comprehensive safety assessments (53). A multi-faceted approach is employed to fulfill this critical requirement. Initially, probiotic strains must undergo meticulous characterization to verify their taxonomic classification, ensure purity, and confirm viability. This process entails a detailed analysis of both genetic and phenotypic attributes (217).

Furthermore, a thoroughly documented history of safe use and precise strain identification are essential prerequisite for ensuring consumer safety. Central to this evaluation is a rigorous assessment of pathogenic potential, including analyses of a strain’s ability to cause infection or produce harmful metabolites (544).

To explore specific physiological risks, such as the ability to provoke endocarditis in vulnerable subjects, studies utilizing animal models are often conducted. In conclusion, exhaustive quality control testing of the final product is indispensable to ensure it is free of hazardous contaminants, including pathogenic microbes, mycotoxins, and heavy metals (544).

### 13.2 Evidence from clinical and epidemiological studies

The safety profile of probiotics has been rigorously evaluated through comprehensive reviews and clinical trials designed to detect potential adverse effects. Evidence accumulated from controlled studies demonstrates no measurable harmful outcomes associated with commonly used strains of *Lactobacillus* and *Bifidobacterium* (545).

Lactic acid bacteria, including genera such as *Lactobacillus*, *Streptococcus*, and *Enterococcus*, have a long history of safe human consumption in fermented foods and dairy products. Despite the fact that certain species exhibit opportunistic pathogenic traits, these microorganisms are generally recognized as safe for dietary use (546). For example, *L. acidophilus* is extensively utilized for immune support and has been applied in managing a spectrum of conditions, ranging from canker sores and hives to digestive disorders and cancer (547).

Prudence remains warranted when administering probiotics to immunocompromised individuals, including neonates, patients with centrally inserted venous catheters, postoperative patients, and those with severe comorbidities. When used at appropriate dosages and in carefully selected candidates, probiotics can provide meaningful therapeutic benefits; however, non-indicated or unregulated consumption may increase the risk of adverse outcomes (548).

Associated risks encompass the generation of deleterious metabolites, the onset of systemic infections, immunopathological responses, and the horizontal transfer of antibiotic resistance genes to indigenous GI microbiota (549–551).

### 13.3 Specific risk pathways

#### 13.3.1 Immune system deviation and hyperactivation

Probiotic supplementation may induce allergic reactions or cause transient GI symptoms, particularly during the initial adaptation phase. Commonly observed adverse effects include abdominal discomfort, bloating, flatulence, and diarrhea, which generally diminish as the host’s system acclimates to the microbial intervention (552).

The gut microbiota plays a pivotal role in immune regulation, supporting innate immune responses, germinal center formation within lymphoid tissues, and the induction of oral tolerance to dietary antigens. Introducing exogenous probiotic strains can modify the homeostatic balance of resident gut microbial communities, potentially eliciting unforeseen immunological effects. Clinical studies have shown that certain *Lactobacillus* strains can enhance interferon-γ production, a key T-helper 1 (Th1) cytokine, while *in vitro* investigations suggest a concurrent suppression of T-helper 2 (Th2) cytokine signaling pathways (553). Notably, excessive amplification of Th1-mediated interferon-γ responses could potentially compromise pregnancy outcomes, including fetal survival rates (553).

#### 13.3.2 Mutagenesis and antimicrobial resistance transfer

Orally administered probiotic strains can transiently colonize the human GI tract, conferring therapeutic benefits. However, these effects are often short-lived and may be offset by the potential risk of introducing antimicrobial resistance determinants (532, 554). Murine model investigations have revealed that probiotics can adapt to host physiological stresses, potentially contributing to the development of antibiotic resistance (555).

A paramount concern involves the role of probiotics—especially those taxonomically related to pathogenic species as potential vectors for the horizontal transfer of antibiotic resistance genes to autochthonous gut microorganisms (556). This risk is particularly significant given the documented intrinsic antibiotic resistance profiles of numerous *Lactobacillus* species, which demonstrate natural resistance to vancomycin, tetracycline, chloramphenicol, and erythromycin. The presence of these chromosomally encoded resistance mechanisms intensifies concerns regarding the dissemination of resistance determinants to more virulent bacterial pathogens, thereby posing a considerable challenge to public health security (556).

#### 13.3.3 Undesirable metabolic events

A key safety concern is the potential of probiotics, particularly those closely related to pathogenic species, to serve as vectors for horizontal transfer of antibiotic resistance genes to indigenous gut microorganisms. This risk is underscored by the intrinsic antibiotic resistance observed in many *Lactobacillus* species, which naturally exhibit resistance to agents such as vancomycin, tetracycline, chloramphenicol, and erythromycin (557).

While supplementation with probiotics can initially disrupt conventional metabolic activities at the intestinal mucosal barrier, potentially inducing broader physiological effects, current research suggests that such alterations are predominantly temporary. These effects commonly diminish once a state of microbial and metabolic equilibrium is reestablished in the GI tract (558).

#### 13.3.4 GI and intestinal side effects

Notwithstanding their well-documented health benefits, the use of probiotic supplements requires careful evaluation, particularly in immunocompromised individuals, pregnant women, critically ill neonates, and patients in postoperative recovery. In these vulnerable populations, administering probiotics carries a risk of serious adverse events, most notably systemic infections. More commonly reported minor side effects include GI disturbances such as abdominal cramping, nausea, flatulence, loose stools, and alterations in taste perception (543, 559).

In a contrasting finding, a clinical study evaluating probiotics for prophylaxis against *C. difficile*-associated diarrhea reported a statistically significant 18–20% lower incidence of these same GI adverse effects in the probiotic group relative to control participants (385).

#### 13.3.5 Virulence trait expression and reversion risk

Of particular concern in the safety evaluation of probiotics is their potential for virulence reversion, a risk that is markedly higher in strains of pathogenic origin, particularly under favorable environmental conditions. This pathogenic potential is intrinsically tied to their ability to adhere to the intestinal mucosa. While mucosal adhesion is indispensable for probiotic colonization and therapeutic efficacy, it concurrently increases the likelihood of bacterial translocation and activation of virulence factors. Consequently, the very mechanisms that enable a probiotic’s beneficial effects may also heighten its capacity to cause disease (560).

Apostolou et al. (560) provided compelling evidence of this relationship, demonstrating a direct link between mucosal adhesion and pathogenic potential in *Lactobacillus* species. Their results revealed that clinical isolates obtained from blood cultures exhibited a significantly higher capacity to adhere to mucosal surfaces than strains isolated from human fecal samples or dairy products. Animal model data provide additional support for the risk of systemic infection. In one study, athymic mice were colonized with human-derived strains of *Bifidobacterium animalis*, *Limosilactobacillus reuteri*, *L. acidophilus*, and *L. rhamnosus* GG (LGG), thereby demonstrating the potential for translocation and systemic spread. While most mice remained asymptomatic, colonization with *L. rhamnosus* GG (LGG) and *L. reuteri* strains led to lethal infections in a subset of immunodeficient neonatal mice (560). These findings indicate that immunocompromised infants may be at considerably heightened risk of probiotic-associated sepsis.

#### 13.3.6 Excessive immunostimulation in compromised hosts

Emerging evidence indicates that probiotic administration can precipitate severe infections and other adverse events in specific patient populations. A major concern is the association with an increased incidence of autoimmune inflammation and related disorders, attributable to the ability of certain probiotic strains to overactivate immune responses. Individuals who are immunocompromised, critically ill, or recovering from surgery appear to be at particularly high risk for these complications (561).

The pathogenic processes involved are multifactorial, encompassing direct impacts on the host and cooperative interactions with indigenous commensal microbiota that can lead to immune dysregulation. A primary objective of subsequent scientific investigation is to decipher the complex interactions among the host, probiotics, and microbiota. Other essential research goals include defining the exact mechanisms of action, unequivocally associating specific probiotic strains with defined health benefits, and establishing the minimum effective concentrations required to produce these therapeutic effects (562).

### 13.4 Regulatory oversight and post-market surveillance

The development of a robust safety profile for probiotics in target demographics is a critical clinical imperative that requires rigorous scientific validation. This process requires meticulous surveillance of adverse events throughout clinical trials and comprehensive safety verification for each individual strain (563). Although international regulatory frameworks differ, universal compliance is essential, mandating that manufacturers satisfy specific safety and efficacy standards established by health authorities for products classified as foods or dietary supplements (563).

Stringent quality control measures are essential throughout the entire manufacturing lifecycle. This requires rigorous verification of product potency, stability, and shelf-life as stated on the label, achieved through strict compliance with good manufacturing practices (GMP) and advanced process controls (564). In addition, a comprehensive post-marketing surveillance system is critical for the ongoing detection and assessment of adverse events, enabling the rapid identification and management of safety concerns that may emerge after commercial release (564).

Effective risk mitigation must prioritize the safety of at-risk populations, including individuals who are immunocompromised and those with significant comorbidities. The paramount importance of safety is further highlighted by the widespread use of probiotics among vulnerable groups, including infants, the elderly, and immunocompromised patients (565).

Core safety evaluations typically encompass strain identification and characterization, pathogenicity and toxicity assessment, and contaminant screening (160). Precise taxonomic identification, confirmation of genetic purity, and verification of viability are fundamental, as consumer safety depends on the use of well-defined strains with a documented history of safe application. Each strain must be rigorously evaluated to confirm the absence of pathogenic traits, such as the ability to cause infection or produce toxins (142). Preclinical models are often employed to assess specific risks, including the potential to induce endocarditis in susceptible hosts (160). Finally, stringent testing of the finished product ensures freedom from hazardous contaminants—including pathogenic microorganisms, mycotoxins, and heavy metals—thereby safeguarding end-product safety (142).

Within the United States, probiotic regulation falls under the authority of the FDA, which generally classifies these products as dietary supplements or food ingredients in accordance with the Dietary Supplement Health and Education Act (DSHEA) of 1994 (566). This regulatory framework does not mandate pre-market approval; instead, it places full responsibility on manufacturers to ensure the safety, quality, and accuracy of labeling before a product is released to the market (566).

### 13.5 Balancing safety with clinical benefit

Among the primary safety concerns associated with probiotic administration are opportunistic infections, as immunocompromised individuals face a significantly increased risk of systemic infections originating from probiotic strains. Notably, cases of *Saccharomyces fungemia* have been reported in critically ill patients and those with central venous catheters, underscoring the need for vigilant risk assessment and monitoring in vulnerable populations (283).

GI disturbances are among the most common side effects of probiotic use, with a subset of consumers experiencing mild, transient symptoms such as bloating, flatulence, or diarrhea at the start of supplementation (567). Potential drug interactions also warrant attention, as probiotics may alter the pharmacokinetics or efficacy of immunosuppressive therapies, thereby increasing infection risk or compromising therapeutic outcomes, posing particular concerns for patients undergoing chemotherapy, organ transplant recipients, and individuals with HIV/AIDS. Moreover, use in vulnerable populations, including neonates and young children, requires careful risk–benefit evaluation to prevent GI complications and ensure safe administration (567).

## 14 Limitations and future perspectives

Despite considerable advancements, significant limitations continue to hinder the interpretation and clinical application of probiotic research. A primary concern is the lack of standardization in product composition, dosing, and quality assurance (567). Independent assessments of commercial probiotics consistently reveal discrepancies between label claims and actual contents, including inaccurate strain designations, missing strain-level information, and viable counts that are either lower or higher than declared, especially in food products (567, 568).

For instance, a 2023 analysis (568) and a 2025 compositional survey (569) documented frequent mislabeling and contamination in multi-strain products. These inconsistencies pose challenges in comparing studies, establishing definitive dose–response relationships, and extrapolating results from rigorously characterized preparations used in clinical trials to the heterogeneous products accessible to consumers (569).

Recent regulatory guidance documents, such as the 2025 Australian Therapeutic Goods Administration (TGA) guidance on the quality of listed probiotic medicines and broader discussions on LBPs from 2020, underscore the necessity for strain-level identification, validated enumeration techniques, and explicit labeling of potency and storage conditions (570, 571). However, the implementation of these standards remains inconsistent across different jurisdictions and product categories (572).

A secondary significant limitation is the pronounced variability in host microbiome composition and clinical responses to identical probiotic interventions. Increasingly, human trials demonstrate distinct responder and non-responder subgroups, even when participants receive the same strain and dosage, with differences correlated to baseline microbial community structures, metabolic profiles, and gut transit characteristics (573). For example, a 2023 RCT revealed that only a subset of adults experienced substantial alterations in gut microbiota and metabolic markers following a standardized probiotic, with responses closely associated with baseline community composition (573). Similarly, 2024 studies assessing *Bacillus*-based probiotics and other formulations indicated that baseline gut microbiome signatures could predict serum amino acid and metabolic responses to supplementation (574).

Extensive cohort and methodological analyses underscore significant inter- and intra-individual variability in gut microbial markers over time, suggesting that a single “snapshot” profile may be inadequate for predicting probiotic responsiveness (575). Nevertheless, many trials are neither designed nor sufficiently powered to classify participants based on baseline microbiome, diet, or host genotype (576). Moreover, only a minority incorporates multi-omics approaches that might yield predictive biomarkers (577). Consequently, the widely held notions of “personalized probiotics” and “precision nutrition” remain aspirational rather than practically applicable within routine settings (578).

A third area of concern pertains to safety assessment and regulatory oversight, particularly for next-generation and genetically engineered strains. A 2023 safety review of probiotics highlighted theoretical and documented risks, including bacteremia or fungemia in high-risk patients, immune dysregulation, and the potential for horizontal transfer of antimicrobial resistance or virulence genes within the gut ecosystem (525).

These concerns are intensified for engineered LBPs, which may incorporate synthetic circuits or therapeutic payloads designed to modulate host pathways. Recent policy analyses and regulatory commentaries—such as a 2025 (579) and previous studies from 2020 to 2024 on LBPs underscore that these products challenge existing food and drug classifications and necessitate customized frameworks that address their living nature, genetic stability, and potential environmental dissemination. Currently, few jurisdictions possess specific standards for clinical trial design, long-term pharmacovigilance, or risk–benefit assessments of genetically modified probiotics (580).

Additionally, recent studies from 2024 to 2025 on NGPs highlight the urgent requirement for clearer guidance and harmonized approval procedures (156, 539).

Addressing these limitations will necessitate coordinated advancements across scientific research, regulatory frameworks, and clinical methodologies. From a research standpoint, extensive, strain-specific multicenter trials that include comprehensive product characterization, standardized outcome measures, and sufficient follow-up are essential to establish minimal effective doses, assess the durability of effects, and identify infrequent adverse events. The integration of metagenomics, metabolomics, and host immunologic and metabolic profiling—approaches that have been increasingly highlighted in human probiotic trials and precision nutrition studies conducted between 2023 and 2025 will be pivotal in understanding the mechanisms that distinguish responders from non-responders and in developing validated predictive biomarkers to inform personalized probiotic selection (581).

Concurrently, regulatory agencies are beginning to issue more detailed guidelines on quality and labeling requirements, and are exploring specific pathways for LBPs and engineered strains. However, additional efforts are required to achieve global consensus and to balance innovation with patient safety (294). Ultimately, advancements in these areas will determine whether probiotics and related live biotherapeutics can transition from broadly categorized ‘‘health-promoting’’ supplements to precisely targeted, mechanism-based interventions that are incorporated into evidence-based guidelines for both prevention and treatment (19, 294).

## 15 Conclusion

Probiotics have emerged as versatile modulators of human health, with evidence supporting their role in GI disorders, metabolic diseases, infections, inflammatory conditions, cancer, and neurological health. Their benefits are mediated through diverse mechanisms, including modulation of the gut microbiota, competitive exclusion of pathogens, regulation of immune and inflammatory pathways, and interactions with host metabolism and the gut–brain axis. At the same time, probiotics are not without limitations: effects are strain-specific, dose-dependent, and influenced by host genetics, diet, and microbiome diversity.

Long-term safety, potential adverse effects, and inconsistent clinical outcomes remain challenges that must be addressed. This review highlights how the probiotic field is advancing beyond traditional applications to encompass NGPs, live biotherapeutics, genetically engineered microbes, and rational microbial consortia. The synergistic potential of probiotics with dietary bioactives, such as fibers and polyphenols, further broadens their scope in precision nutrition and targeted therapy.

Unlike earlier reviews, this synthesis integrates historical context, current applications, mechanistic insights, safety considerations, and future prospects to provide a holistic perspective. Moving forward, well-designed clinical trials, standardized protocols for strain selection and dosage, and regulatory harmonization will be essential for translating laboratory findings into effective interventions. With these advances, probiotics have the potential to shape the next generation of functional foods and microbiome-based therapeutics, contributing significantly to human health and precision medicine.
